# Knowledge of Dietitians on Gut Microbiota in Health—An Online Survey of the European Federation of the Associations of Dietitians (EFAD)

**DOI:** 10.3390/nu16050621

**Published:** 2024-02-23

**Authors:** Evdokia K. Mitsou, Christina N. Katsagoni, Katarzyna Janiszewska

**Affiliations:** 1 Department of Nutrition and Dietetics, School of Health Science and Education, Harokopio University, 17671 Athens, Greece; 2 Department of Clinical Nutrition, Agia Sofia Children’s Hospital, 11527 Athens, Greece; christina.katsagoni@gmail.com; 3 The European Federation of the Associations of Dietitians (EFAD), Gooimeer 4-15, 1411 DC Naarden, The Netherlands; katarzyna.janiszewska@efad.org

**Keywords:** online survey, dietitians, knowledge, gut-health interconnection, gut microbiota manipulation, probiotics, prebiotics

## Abstract

Explorations of current knowledge of dietitians about gut-health interconnection and the role of diet in gut microbiota manipulation are rather scarce in the literature. In this online survey we assessed the perceived and current knowledge of dietitians across Europe about gut microbiota and systemic health, nutrition as a modulator of the gut ecosystem, and the role of probiotics and prebiotics. Pre-graduate dietetic students and other professionals were also invited to participate. A total of 179 full responses were recorded (dietitians, n = 155), mainly from Southern and Western regions. Most participants (>78.0%) reported an average to good level of perceived knowledge, with significant positive correlations between perceived and current knowledge in all sections and overall (*p* for all <0.05). Nevertheless, a rather low current knowledge scoring of participants about probiotics and prebiotics was observed. Features such as being a dietitian, having a higher educational level as dietitian and working in an academic/research setting were usually associated with higher current knowledge. Further analysis revealed that dietitians had a trend for higher scoring about probiotics and prebiotics compared to pre-graduate students or other professionals. Moreover, for dietitians, working in an academic or research setting was an independent factor for scoring in the highest quartile in all tested sections and overall (*p* for all <0.05). In conclusion, this online survey shed some light on the current knowledge of dietitians across Europe about gut microbiota parameters, including dietary modulation, highlighting in parallel possible knowledge determinants. Potential areas for future educational efforts in this rather unexplored field were indicated.

## 1. Introduction

Gut health constitutes an integral part of overall human health. Beyond its fundamental role in digestion and absorption of nutrients from food, the gut is the largest immune and endocrine organ in the human body, which interconnects extensively with other organs through several axes and may affect host metabolic and mental health [1]. Moreover, the gut barrier is a multi-layer functional unit that serves as a mechanical, immunological, and biological line of health defence. Various gastrointestinal diseases [e.g., inflammatory bowel disease (IBD), irritable bowel syndrome (IBS), and celiac disease] have been linked to gut barrier dysfunction. The latter is also well correlated with acute and chronic disease states, including autoimmune, inflammatory, and metabolic disorders (e.g., obesity and diabetes) [2,3].

Gut microbiota, the unique collection of bacteria, archaea, fungi, and viruses mainly located in the large intestine, has been recently highlighted as a key player in gut interactions and interconnections [4,5]. Though the definition of a healthy gut microbiota is still rather inconclusive, a growing body of evidence is documenting the multifaceted role of gut microbiota characteristics in human physiology and health status [6]. Host or lifestyle factors might affect the gut microbiota composition and function throughout the life cycle, with nutrition being characterized as a fundamental contributor to gut microbiome dynamics [7]. Dietary components, food choices, and dietary patterns have a major role in shaping gut microbial profile and metabolic activity, with potential short or long-term effects on human health [7]. Furthermore, the role of probiotics and prebiotics as modulators of gut microbiota and their beneficial contribution in gastrointestinal and overall health have been extensively explored [8].

Prior online surveys are available regarding evaluation of the knowledge of healthcare professionals, including dietitians, surrounding the use of probiotics and prebiotics [9,10,11,12,13,14]. Nevertheless, the exploration of current knowledge of dietitians about gut-health interconnection, gut microbiota parameters, and the role of diet in gut microbiota manipulation is rather scarce. Under this context, the aim of this cross-sectional study was to evaluate through an online survey the level of knowledge and possible educational needs of dietitians across Europe about gut microbiota and systemic health and the role of nutrition and diet as modulators of the gut ecosystem. Feedback from pre-graduate dietetic students and other professionals was also included. The effect of potential determinants (i.e., European region, age group, educational level, professional background) on tested variables was further analysed.

## 2. Materials and Methods

### 2.1. Subjects

The survey was an initiative of the European Federation of the Associations of Dietitians (EFAD) and was addressed to dietitians and pre-graduate dietetic students around 28 countries with National Dietetic Associations (NDAs) members of the EFAD. Other professionals interested in the field of gut microbiota were also invited to the study. The 28 countries included in the survey (Austria, Belgium, Croatia, Cyprus, Czech Republic, Denmark, Finland, France, Germany, Greece, Hungary, Iceland, Ireland, Israel, Italy, Lithuania, Luxembourg, Romania, The Netherlands, Norway, Poland, Portugal, Slovenia, Spain, Sweden, Switzerland, Turkey, and the United Kingdom) were further grouped into one of the four European regions (Central and Eastern, Northern, Southern, and Western Europe) based on EuroVoc, with Turkey and Israel being included in the Southern region (available at: https://eur-lex.europa.eu/browse/eurovoc.html?params=72,7206,911#arrow_911, accessed on 1 February 2021).

### 2.2. Survey Questionnaire

The content of survey questions was inspired by the scientific expertise of the survey project officer (Ε.K.Μ.) in the field of gut microbiota and nutrition and the Advisory Board members of EFAD, as well as previous surveys regarding the knowledge of health care professionals/dietitians about probiotics and prebiotics [9,10,11,12,13,14], the guidelines of organizations and associations regarding the use of probiotics and prebiotics [15,16,17,18,19,20], clinical guides about probiotic supplements available in Canada and the United States of America (http://www.probioticchart.ca/; http://usprobioticguide.com/, both accessed on 18 January 2021), and web-based sources about gut microbiota and nutrition [Gut Microbiota for Health (https://www.gutmicrobiotaforhealth.com); International Probiotics Association (https://internationalprobiotics.org); International Probiotics Association Europe (https://www.ipaeurope.org/); International Scientific Association for Probiotics and Prebiotics (ISAPP) (https://isappscience.org); United European Gastroenterology (https://ueg.eu)] (all accessed on 21 January 2021).

Questions were formulated by the survey project officer in collaboration with the Advisory Board members of the EFAD. A pre-test of the survey was conducted during March 2021 with a group of 21 experts and non-experts in the gut microbiota field, 14 professional dietitians included, to identify possible weaknesses of the questionnaire and to detect technical issues regarding the online survey tool. Pre-test feedback contributed to the finalization of the questionnaire, resulting in a total number of 77 survey questions and nine sections covering different aspects of health through the gut (Supplementary Materials File S1—EFAD surveys-Health through Gut). The survey included sections about sociodemographic data (country, age group, educational level, workplace, and years in practice as a dietitian) and the perceived and current knowledge of participants in four different fields (gut health and overall health, nutrition as a gut microbiota modulator, probiotics, and prebiotics). Sections about beliefs, attitudes, dietary practices, and future educational aspects were further included in the survey, beyond the scope of this report.

In detail, the survey assessed the participants’ perceived and current level of knowledge in four different areas (the role of gut health in overall health, the role of nutrition as a gut microbiota modulator, the role of probiotics in health, and the role of prebiotics in health). Perceived knowledge for each section was reported by the participants using a five-point Likert scale (no knowledge, poor knowledge, average knowledge, good knowledge, excellent knowledge). A mean overall perceived level of knowledge was also estimated based on the values across the four sections. Current knowledge was scored based on the “true/false” and multiple-choice questions provided for each section (22, 11, 12 and seven questions, respectively). A total score of current knowledge, based on the 52 questions across the four sections, was also provided.

The survey was conducted online using LimeSurvey (Version 3.5.4+ 180320), a web server-based software for EFAD surveys (http://www.efadsurveys.eu/index.php/485711?lang=en) (accessed on 22 April 2021). Participants were asked to consent before completing the survey. Participation in the survey was completely voluntary. Initially, the IP address of the respondent was collected to automatically exclude any attempts to fill out the survey multiple times from the same IP. After exclusion, data were cleaned, and no identifying information was further associated with the responses. There was no other identifying information collected. The responses were processed in anonymised form (extracted from the survey platform) and were kept confidential.

### 2.3. Survey Communication Plan

The survey was officially launched through the quarterly distributed EFAD Newsletter (Newsflash) on 22 April 2021. Communication efforts were implemented as an educational hub with other surveys and individually through several communication channels [Newsflashes (n = 3); social media of the EFAD, NDAs, and the European Network of Dietetic Students (ENDietS); members of EFAD expert networks; personal contacts of the project officer and Advisory Board members]. Reminders were sent in a targeted mode based on the participation feedback throughout the study. The data collection period for analysis lasted from 22 April 2021 until 22 July 2021.

### 2.4. Statistical Analysis

Data were presented as median (Q1–Q3) for continuous variables, and frequencies (n, %) for categorical variables. The normality of distribution of continuous variables was tested using the Shapiro-Wilk test. For continuous variables, comparisons among and between groups were performed based on non-parametric tests (Kruskal-Wallis and Mann-Whitney U tests, respectively), whereas cross tabulation analysis of categorical variables was performed using a chi-squared (X^2^) test. Correlation analysis (Spearman’s rho) was further applied, where applicable. Multiple logistic regression analysis was applied for score quartiles of current knowledge to identify significant independent explanatory variables of this parameter (i.e., European region, age group, educational level, professional background). The significance level was set at 5.0% (*p* < 0.05). Statistical analysis was performed by Stata 15.1 [21]. Based on the total number of dietitians/members of EFAD (35,000.00 approximately), sample size calculation indicated at least 380 responses to have a representative sample in a 95.0% confidence interval.

## 3. Results

### 3.1. Responses

During the data collection period (22 April 2021–22 July 2021), a total of 2087 visits to the survey platform were recorded. Exclusion of multiple attempts from the same IP, at the same date and proximal recorded time, with no further information (n = 426), resulted in 1661 responses. From these 1661 participants, 290 started to complete the survey (17.5% of those interested in the study) and, finally, 179 participants provided full responses (61.7% completion rate; 10.8% of those interested in the study). Data analysis was based on full responses (N = 179), with all sections data available.

### 3.2. Sociodemographic Data (Country/Region—Participants’ Characteristics)

Of the 179 participants that provided full responses during the study period, 155 were dietitians, 15 pre-graduate dietetic students, and nine other professionals. Most of the full responses resulted from the Southern (55.3%) and Western European (25.1%) regions (Table 1; Supplementary Materials Figure S1). More than 60.0% of participants were under the age of 35, whereas 56.2% of the dietitians had a MSc or PhD degree, and most of them had a maximum of four years in dietetic practice. Discrepancies in participants’ characteristics were detected among regions. In Western Europe, participants were older and more experienced dietitians compared to the Central-Eastern and Southern regions (*p* for all <0.05). Nevertheless, dietitians in Western Europe more frequently reported a basic degree of dietetic education (BSc or BTS) compared to Southern Europe, where dietitians were more frequently characterized by a higher educational level (MSc/PhD) (*p* = 0.022). Compared to participants from Southern region, dietitians from the Northern region were more frequently employed in clinical settings (71.4% vs. 22.7%), but less frequently in academia/research (7.1% vs. 29.5%) or as freelancers (0.0% vs. 33.0%) (*p* = 0.001).

### 3.3. Level of Knowledge about Gut Health in Overall Health, Nutrition as a Gut Microbiota Modulator, and Probiotics/Prebiotics in Health

#### 3.3.1. Section: The Role of Gut Health in Overall Health

Most of the participants (80.5%) reported an average to good level of perceived knowledge about the role of gut health in overall health, regardless of being a dietitian, a student, or other professional (Table 2). Dietitians with higher educational levels reported higher perceived knowledge compared to those with only undergraduate studies (overall *p* < 0.001), whereas participants working in an academic/research setting had greater perceived knowledge compared to freelancers (*p* = 0.044).

For all participants (N = 179) and dietitians only (n = 155), the median score of current knowledge in this section was 18.00 (Q1–Q3: 16.00–20.00) out of 22 questions (Table 3). No significant overall differences in median scores were detected among European regions, age groups, dietitians/students/other professionals, or years in practice as a dietitian. Working in an academic/research setting was characterized by a significantly higher score compared to working in community, industry, or being a freelancer (*p* for all <0.05), whereas dietitians with a PhD or a MSc degree trended towards higher scoring (Table 3). Furthermore, a significant positive correlation was observed between perceived and current knowledge for all participants (N = 179; Spearman’s rho 0.353, *p* < 0.001) and dietitians only (n = 155; Spearman’s rho 0.372, *p* < 0.001) (Supplementary Materials Figure S2a,b).

**Figure 1:**
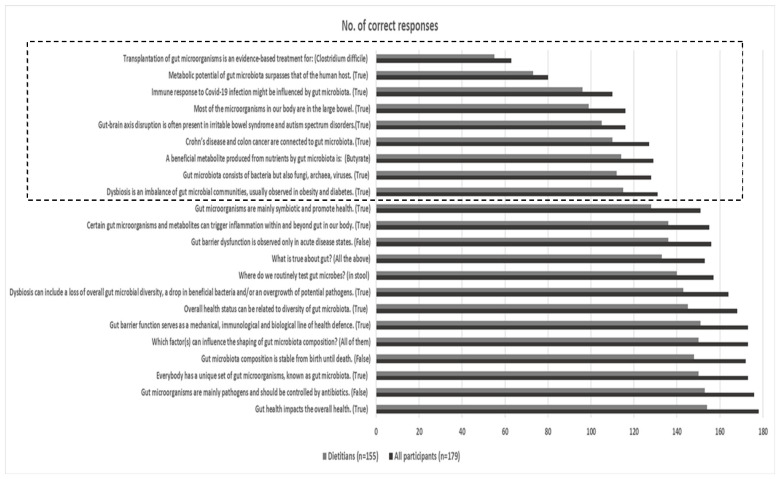
Number of correct responses for all participants (N = 179) and dietitians (n = 155) for the 22 questions included in the section about the role of gut health in overall health. The dotted frame indicates questions with a more limited rate of correct responses.

The number of correct responses for each of the 22 questions included in this section are provided in Figure 1. Correct response rates were similar in the overall (N = 179) and the dietitians group (n = 155). A high rate of correct responses was detected in questions regarding the role of gut health and gut barrier in overall health, and some gut microbiota parameters, including composition (i.e., uniqueness, diversity, stability, factors affecting shaping), dysbiosis, microbes as symbiotes/pathogens, stools as a test matrix for gut microbiota, and the role of gut microbiota in inflammation. On the contrary, a more limited rate of correct responses was observed in the case of other gut microbiota characteristics such as location and composition, metabolic potential, contribution to disease states or faecal transplantation as a therapeutic mean, suggesting potential areas for further education.

#### 3.3.2. Section: The Role of Nutrition as Gut Microbiota Modulator

Most of the participants (83.0%) reported an average to good level of perceived knowledge about the role of nutrition and diet as gut microbiota modulators, regardless of being a dietitian, a student, or other professional; in terms of workplace (*p* = 0.046), participants working in academic/research settings tended to have higher perceived knowledge compared to freelancers (*p* = 0.076) (Table 2).

A significant positive correlation was observed between perceived and current knowledge for all participants (N = 179; Spearman’s rho 0.454, *p* < 0.001) and dietitians only (n = 155; Spearman’s rho 0.447, *p* < 0.001) (Supplementary Materials Figure S3a,b). The median score of current knowledge in this section of 11 questions was 8.00 (6.00–9.00) and was the same for all participants (N = 179) and dietitians only (n = 155) (Table 4). Dietitians (*p* = 0.003) and other professionals (*p* = 0.007) had higher actual knowledge in this section compared to pre-graduate dietetic students. Moreover, the level of knowledge was higher in the Western compared to the Southern region (*p* = 0.046), possibly due to the higher participation of students in the Southern area. Dietitians with a BSc (*p* = 0.052), MSc (*p* = 0.033), or PhD (*p* = 0.006) had higher current knowledge compared to those pre-BSc, whereas dietitians with a PhD tended to have higher scores compared to those at a BSc (*p* = 0.094) or MSc (*p* = 0.085) educational level. Furthermore, working in an academic/research setting was characterized by higher scoring compared to working in community service (*p* = 0.021), other settings (*p* = 0.021), or being a freelancer (*p* = 0.011) (Table 4), with a similar trend observed for clinical settings compared to community service (*p* = 0.080).

**Figure 2:**
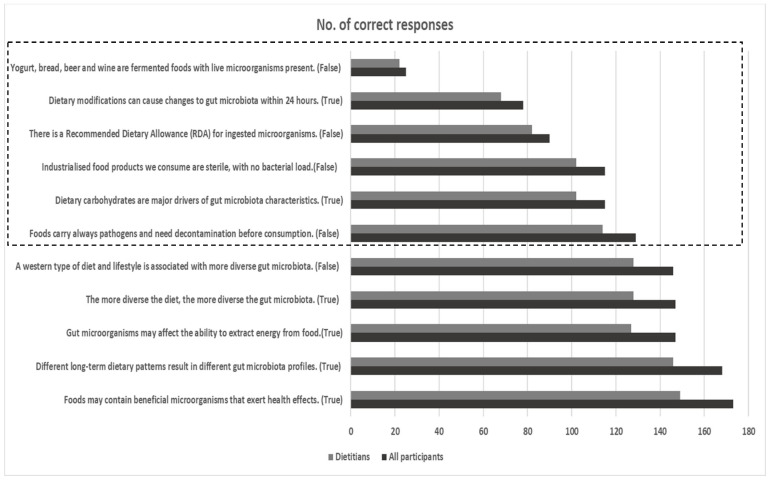
Number of correct responses for all participants (N = 179) and dietitians (n = 155) for the 11 questions included in the section about the role of nutrition as a gut microbiota modulator. The dotted frame indicates questions with a more limited rate of correct responses.

Similar rates of correct responses were detected in the overall (N = 179) and dietitian group (n = 155) (Figure 2). Most participants recognized the role of foods as vehicles for beneficial microbes, the contribution of long-term dietary patterns and diet diversity to gut microbiota shaping, and the potential of intestinal microbes to extract energy. On the contrary, correct responses were more limited when it came to questions regarding fermented foods as vehicles for live microorganisms, the role of food processing and decontamination on bacterial load, the Recommended Dietary Allowance (RDA) for ingested microorganisms, and the short-term/long-term effects of dietary modifications and nutrients on gut microbiota. For instance, only 14.2% of dietitians correctly answered the question about the presence of live microorganisms in fermented foods such as yogurt, bread, beer, and wine, highlighting potential educational gaps in this section.

#### 3.3.3. Section: The Role of Probiotics in Health

Most participants (78.8%) reported an average to good level of perceived knowledge about the role of probiotics in health, regardless of being a dietitian, a student, or other professional (*p* = 0.290) (Table 2). Dietitians with 20 or more years of working experience had higher perceived knowledge about probiotics compared to those less experienced (0–4 years; *p* = 0.045), whereas participants in the age groups of 30–34 or 50–54 reported higher perceived knowledge compared to some other age groups (overall *p* = 0.054).

The section about the role of probiotics in health had 12 questions for current knowledge scoring, including the definition of probiotics. The median score of current knowledge in this section was 5.00 (Q1–Q3: 4.00–7.00) for all participants, with a median value of 6.00 in the case of dietitians only (Table 5). A significant positive correlation was observed between perceived and current knowledge for all participants (N = 179; Spearman’s rho 0.362, *p* < 0.001) and dietitians only (n = 155; Spearman’s rho 0.361, *p* < 0.001). Nevertheless, participants with an excellent level of perceived knowledge achieved a median score of 6.00 (half of the maximum score value of 12) (Supplementary Materials Figure S4a,b) and 52.0% of all participants had less than six correct responses, a fact that indicated rather low scoring of participants in this section. In contrast to perceived knowledge in this field, no significant overall differences were detected in the current level of knowledge among age groups or years in practice as a dietitian. Parameters such as being a dietitian (vs. students, *p* = 0.052), having a PhD level of education as dietitian (vs. pre-BSc, *p* = 0.035; vs. BSc, *p* = 0.047; vs. MSc, *p* = 0.080), or working in an academic/research setting (vs. industry, *p* = 0.041; clinical setting, *p* = 0.069; freelancers, *p* = 0.088; ‘other’ category, *p* = 0.004) were characterized by higher scores in the probiotic section compared to other groups (Table 5).

**Figure 3:**
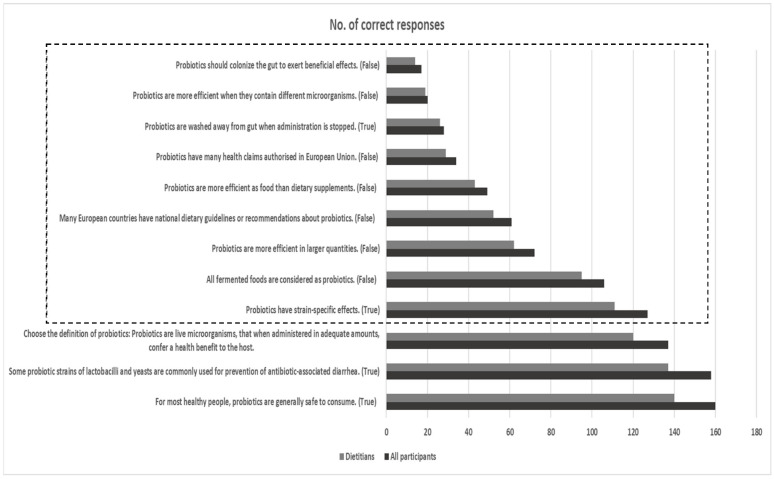
Number of correct responses for all participants (N = 179) and dietitians (n = 155) for the 12 questions included in the section about the role of probiotics in health. The dotted frame indicates questions with a more limited rate of correct responses.

Further analysis based on the number of correct responses in each of the 12 questions included in this section is provided in Figure 3. Similar rates of correct responses were detected in the overall (N = 179) and dietitian (n = 155) group. Most participants (>88.0%) recognized the safe use of probiotics for most healthy people and the common use of some probiotic strains for the prevention of antibiotic-associated diarrhoea (AAD). Furthermore, 77.0% of participants recognized the correct definition of probiotics as “live microorganisms that when administered in adequate amounts can confer a health benefit”. Nevertheless, correct responses were more limited regarding properties and efficacy factors of a probiotic strain, ways of probiotics administration, and questions about legislation, health claims, and national dietary guidelines about probiotics.

#### 3.3.4. Section: The Role of Prebiotics in Health

Most participants (78.2%) reported an average to good level of perceived knowledge about the role of prebiotics in health, regardless of being a dietitian, a student, or other professional (Table 2). Participants in Central and Eastern Europe reported a significantly higher level of perceived knowledge in this section compared to the other regions (*p* for all <0.05); no further overall differences were detected based on participants’ characteristics.

The section about the role of prebiotics in health had seven questions for scoring of current knowledge, including the definition of prebiotics (Table 6). For all participants (N = 179), the median score of actual knowledge in this section was 3.00 (Q1–Q3: 3.00–4.00), with a median value of 4.00 in the case of dietitians only (n = 155). Participants with an excellent level of perceived knowledge achieved a median score of 4.00 (approximately 60.0% of questions correct) and 51.4% of all participants had less than four correct responses, a fact that indicated rather low scoring of participants in this section. Nevertheless, a significant positive correlation was observed between perceived and current knowledge for all participants (N = 179; Spearman’s rho 0.351, *p* < 0.001) and dietitians only (n = 155; Spearman’s rho 0.347, *p* < 0.001) (Supplementary Materials Figure S5a,b). In contrast to the perceived knowledge, no significant overall differences in current level of knowledge about prebiotics were detected among European regions (*p* = 0.620). Parameters such as being a dietitian (vs. students, *p* = 0.041), having a PhD (vs. pre-BSc, *p* = 0.008; vs. BSc *p* = 0.010) or a MSc (vs. pre-BSc, *p* = 0.033) level of education as a dietitian, working in an academic/research setting (vs. all other settings, *p* for all <0.05), or having 20 or more years in practice (vs. 0–4 yrs: *p* = 0.061; vs. 10–19 yrs: *p* = 0.063) were characterized by higher scores in the prebiotics section compared to other groups.

**Figure 4:**
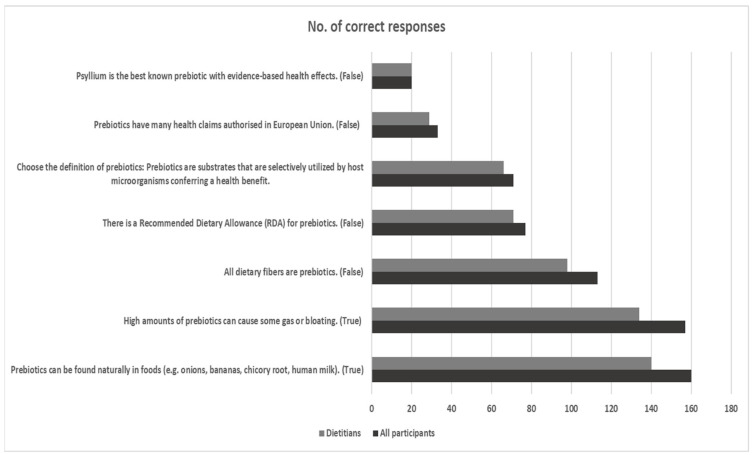
Number of correct responses for all participants (N = 179) and dietitians (n = 155) for the 7 questions included in the section about the role of prebiotics in health. The dotted frame indicates questions with a more limited rate of correct responses.

Similar rates of correct responses were detected in the overall (N = 179) and dietitian (n = 155) groups (Figure 4). Most participants (>86.0%) recognized foods as natural sources of prebiotics and the side-effects of high prebiotic intake. Only 63.0% of participants and dietitians recognized that not all dietary fibres are prebiotics. Furthermore, four out of 10 participants/dietitians reported the correct definition of prebiotics (“prebiotics are substrates that are selectively utilized by host microorganisms conferring a health benefit”), with most incorrect answers related to prebiotics being dietary fibres. Furthermore, four out of 10 participants/dietitians reported the lack of an RDA for prebiotics, two out of 10 participants/dietitians provided a correct response regarding health claims, whereas only one out of 10 dietitians answered correctly about psyllium not being the best-known prebiotic, with no other participant group (students, other professionals) providing the correct answer.

#### 3.3.5. Overall Perceived Knowledge and Total Score of Knowledge

Most participants (83.0%) reported an average to good level of overall perceived knowledge, based on the four sections. Participants aged 45–49 yrs and dietitians with 20 or more years in practice had higher perceived knowledge (Table 2). The median total score for current knowledge, evaluated by all 52 questions, was 35.00 (Q1–Q3: 29.00–38.00) for all participants (N = 179), with a median value of 35.00 (Q1–Q3: 30.00–39.00) in the case of dietitians only (n = 155) (Table 7). Perceived and current knowledge were in agreement, as a median score of 35.00 reflected a perceived knowledge of “average to good” in a five-point Likert scale. In addition, a significant positive correlation was observed between overall perceived and total current knowledge for all participants (N = 179; Spearman’s rho 0.459, *p* < 0.001) and dietitians only (n = 155; Spearman’s rho 0.460, *p* < 0.001) (Supplementary Materials Figure S6a,b). Participant characteristics, such as being a dietitian (vs. students, *p* = 0.009), having a PhD (vs. pre-BSc, *p* = 0.009; vs. BSc, *p* = 0.019; vs. MSc, *p* = 0.062) or a MSc (vs. pre-BSc, *p* = 0.028) level of education as dietitian, or working in an academic/research setting (vs. all other working settings, *p* for all <0.05) was characterized by higher scores compared to other groups.

For multiple logistic regression analysis, scoring in each section and total score were ranked into quartiles for all participants and dietitians only. For all participants, analysis for European regions, age groups, and status in the same multiple logistic regression model revealed that dietitians had a trend for scoring six times more in the highest quartile in the probiotics (OR: 6.758, CI: 0.856–53.353, *p* = 0.070) and prebiotics sections (OR: 6.462, CI: 0.840–49.721, *p* = 0.073) compared to students and other professionals. Moreover, being of a younger age (20–24 yrs) was an independent factor determining a 72% lower chance of scoring in the highest quartile in the probiotics section compared to other age groups (OR: 0.278, CI: 0.078–0.987, *p* = 0.048). For dietitians, analysis for variables such as European regions, age groups, educational level, workplace, and years in practice in the same model revealed that working in an academic or research setting was an independent factor determining a two times greater chance of scoring in the highest quartile in the gut health (OR: 2.367, CI: 1.088–5.151, *p* = 0.030) and probiotics sections (OR: 2.205, CI: 1.026–4.735, *p* = 0.043), a three times greater chance of scoring in the highest quartile in the nutrition as a gut microbiota modulator (OR: 2.929, CI: 1.308–6.560, *p* = 0.009) and prebiotics sections (OR: 2.725, CI: 1.205–6.159, *p* = 0.016), and a four times greater chance of being in the highest quartile for overall scoring (OR: 3.898, CI: 1.660–9.156, *p* = 0.002) compared to the other survey workplace choices. Furthermore, being a dietitian from the southern part of Europe was an independent factor determining an approximately 60% lower chance for scoring in the highest quartile in the section about nutrition as gut microbiota modulator (OR: 0.416, CI: 0.201–0.860, *p* = 0.018) and in total score (OR: 0.392, CI: 0.176–0.875, *p* = 0.022) compared to other tested regions.

## 4. Discussion

Online surveys have emerged as a valuable time- and cost-effective tool for field research at the national and trans-national level [22]. In this online survey, we assessed the level of knowledge of dietitians across Europe on gut microbiota and overall health, the role of nutrition and diet as modulators of the gut ecosystem, and the role of probiotics and prebiotics in health and disease. Pre-graduate dietetic students and other professionals were also invited to participate. A total of 179 full responses were recorded (dietitians, n = 155), mainly from the Southern and Western regions of Europe.

Most participants (>78.0%) reported an average to good level of perceived knowledge, with significant positive correlations between perceived and current knowledge in all sections and overall (*p* for all <0.05). Nevertheless, there was a rather low scoring trend for participants’ current knowledge in the sections about probiotics and prebiotics. Characteristics usually associated with higher current knowledge included being a dietitian, having a higher educational level as a dietitian, and working in an academic/research setting. Further analysis revealed that dietitians had a trend towards scoring six times more often in the highest quartile in the sections about probiotics and prebiotics compared to students and other professionals, whereas, for dietitians, working in an academic or research setting was an independent factor that determined an increased likelihood of scoring in the highest quartile for all tested sections and overall (*p* for all <0.05). On the contrary, being of a younger age or a dietitian from the Southern part of Europe was an independent factor for scoring less often in the highest quartile in the probiotics section or the nutrition as a gut microbiota modulator section, respectively.

Participant knowledge about the role of gut health in overall health and some gut microbiota characteristics (i.e., uniqueness, diversity, stability, factors affecting shaping, dysbiosis) was adequate in the present study. However, potential areas for further education were revealed, based on questions with a more limited rate of correct responses, such as those regarding that beyond the recognized presence of bacteria, gut microbiota is also comprised by other members located in the large intestine, namely archaea, fungi, and viruses [6]. Furthermore, participants tended to underestimate the metabolic potential of gut microbiota and subsequently not recognize the multiple implications of metabolic products and structural components of gut microbes on overall health status. In fact, metabolites produced by gut microbiota during colonic fermentation or biotransformation of dietary nutrients and endogenous host components (e.g., short-chain fatty acids, trimethylamine N-oxide, tryptophan and indole derivatives, polyphenols byproducts, secondary bile acids etc.) have a central role in the intra- and extra-intestinal manifestations of gut microbiota in human health [23], whereas bacterial structural characteristics, such as lipopolysaccharides, are involved in inflammatory and immune-related cascades related to host metabolism [24]. Furthermore, education about microbial dysbiosis, gut-barrier malfunction, and disruption of the gut-brain axis need further educational emphasis due to their implications in several disease states, both gastrointestinal and systemic ones [25,26], with most recent data suggesting the potential role of gut microbiota characteristics in individual susceptibility and recovery parameters during COVID-19 infection [27]. Moreover, though rather familiar with faecal microbiota transplantation (FMT) as a term, two out of three dietitians had misconceptions about evidence-based applications of the procedure, such as in the case of recurrent *Clostridium difficile* infection [28]. Dietary choices of both the stool donor and recipient could be a predictor factor for FMT outcome. Considering that FMT is emerging as a potential treatment option for other gastrointestinal and non-communicable diseases in the future [29], the role of dietitians in this area should be further enhanced. Overall, further education about the metabolic machinery of gut microbes and the potential implications of the gut microbiome for health physiology is necessary for dietitians to fully elucidate the ways that the gut microbiota might affect overall health.

Regarding knowledge about the role of nutrition and diet as gut microbiota modulators, dietitians recognized the role of foods as vehicles for beneficial microbes, but further education is necessary about the effects of food processing and decontamination on bacterial load and microbial control of processed foods [30]. Furthermore, participants were familiar with the importance of long-term dietary patterns and diet diversity in gut microbiota shaping and the potential of intestinal microbes to extract energy [31,32], whereas future educational efforts could further decipher the short-term, alongside long-term, effects of dietary modifications on gut microbial dynamics, and the variable impact of macronutrients and micronutrients on gut microbiome characteristics [32,33].

Our data also highlighted the potential educational gaps surrounding fermented foods and their role as vehicles for live microorganisms; only 14.2% of dietitians responded correctly that not all fermented foods are carriers of live microorganisms. Fermented foods and beverages have been a part of food traditions around the world for centuries, and they have been recently defined as “foods made through desired microbial growth and enzymatic conversions of food components” in the respective consensus paper by ISAPP [34]. Fermented foods are classified according to the presence (e.g., yogurt, kefir, most cheeses, non-heated fermented sausages etc.) or absence (e.g., bread, wine, most beers etc.) of live and active microorganisms in the final product, and this classification could serve as a basis for the proper labelling and characterization of fermented foods [34]. Fermented foods could exert intestinal, immune, and systemic health benefits through nutritive modification of the food matrix, the presence of bioactive compounds and metabolites, or modulation of gut microbiota composition and activity [34]. In addition, consumption of live bacterial cultures in yogurt has an approved health claim by the European Food and Safety Authority (EFSA) due to improvement of lactose digestion in the gut [35]. Nevertheless, no RDA for ingested microorganisms is available yet and a better understanding of the health benefits of various available fermented foods through well-designed human clinical studies is further necessary [34,36,37]. Furthermore, “fermented foods” and “probiotics” cannot be used as interchangeable terms, since few available fermented foods contain well-characterized strains with documented probiotic properties [34,38]. All of the above aspects should be taken under consideration in future educational efforts, as they represent important knowledge for proper evidence-based nutritional counselling and consumer awareness about fermented foods.

Probiotics are defined as “live microorganisms that when administered in adequate amounts can confer a health benefit” [38]. Based on this definition, the suggested qualification criteria for probiotics include sufficient strain characterization, safety for intended use, documented health benefit(s) by at least one human clinical trial according to generally accepted scientific standards, and viability at an efficacious dose throughout shelf life of food or dietary supplement product [39]. In this cross-sectional study, 77.0% of all participants and dietitians recognized the correct definition of probiotics, a percentage quite comparable to previous studies among health care providers around the world [9,12,13,40], but higher compared to other dietitian groups in the past [12]. Participants (>88.0%) also recognized the safe use of probiotics for most healthy people and the common use of some probiotic strains of lactobacilli and yeasts for the prevention of AAD in concordance with data from previous surveys [9,11,13].

Though most participants in the present study rated their perceived knowledge about probiotics as average to good, in line with previous data from healthcare professionals in Europe and beyond [9,10], we have noticed misconceptions about properties of probiotic strains, factors of efficacy, and facts related to the legislation framework, health claims, and national dietary guidelines about probiotics in Europe. Mismatches between perceived knowledge of participants and correct answers about probiotics have also been reported in the literature [9]. In contrast to the beliefs of most participants in our study, available data show that gut colonization is not a prerequisite for probiotics to confer health benefits, and that generally probiotics have a short intestinal persistence time and eventually are washed away from the gut after the end of consumption [41,42]. Moreover, the efficacy of probiotics is strain-specific and disease-dependent, whereas the variable efficiency of probiotics has been proposed according to dosage, single-strain or multi-strain form, duration of administration, and way of ingestion (food vs. supplements) in various health conditions [43]. According to European Union (EU) regulation, no probiotic health claim for individual strains has been scientifically approved for product labelling or advertising by EFSA so far; the term “contains probiotics” is considered an example of a health claim, which is not yet authorized by the European Commission [9,44]. Furthermore, the inclusion of probiotics as part of national dietary guidelines or clinical recommendations is so far rather scarce in countries inside and outside Europe [45,46]. Thus, further educational efforts are necessary for nutrition professionals to disentangle the above aspects in the field of probiotic use and legal framework in Europe.

Several surveys are available in literature about probiotics knowledge, use and attitudes among consumers, students, or healthcare professionals; nevertheless, similar data regarding prebiotics are rather limited. Previous feedback from consumers or college students has highlighted limited familiarity with the prebiotic concept, or possible misconceptions in the field [47,48,49]. Regarding knowledge about the role of prebiotics in health, over 86.0% of participants and dietitians in this study recognized the role of foods as natural sources of prebiotics and the possible side-effects of high prebiotic intake, such as gut flatulence and bloating [50]. Prebiotics are defined as “substrates that are selectively utilized by host microorganisms conferring a health benefit” [51] and, based on this definition, the concept of prebiotics includes fermentable dietary fibres and other non-carbohydrate substances with prebiotic potential (i.e., polyphenols, polyunsaturated fatty acids) [51]. In this cross-sectional study, 40.0% of all participants and dietitians recognized the correct definition of prebiotics. Most incorrect definition answers related to prebiotics being exclusively dietary fibres, whereas 63.0% of participants and dietitians recognized that not all dietary fibres are prebiotics. Misconceptions about dietary fibres and prebiotics could be attributed merely to marketing advertisement of commercial products containing prebiotics as “containing fibres” [11]. Nevertheless, the rather low scoring of participants in this section revealed some further misconceptions about prebiotics. For instance, only one out of 10 dietitians answered that the dietary fibre psyllium is not the best-known prebiotic, with no other participants providing the correct answer. In fact, inulin-type fructans and galacto-oligosaccharides are the most recognized and investigated prebiotics, with documented health effects [52], whereas the soluble fibre psyllium is best known as a bulk-forming agent used for the treatment of constipation [53]. Contrary to dietary fibres, no RDA has been established yet for prebiotics, though a dose of around 5 g of prebiotics is advisable by ISAPP to be included in the diet daily (available at: https://isappscience.org/for-scientists/resources/prebiotics/, accessed on 1 May 2022). In addition, to date, only the prebiotic chicory inulin has received an authorized EU health claim surrounding improvement of bowel function [54].

Further data analysis into knowledge levels in our study revealed a good agreement between overall perceived and current knowledge of participants/dietitians in the gut microbiota field across Europe. A previous study of mixed background participants from the United Arab Emirates [55] indicated that being a university student and a healthcare professional were the only significant predictors regarding microbiota knowledge, whereas being a postgraduate participant or a healthcare professional were the only significant predictors for probiotics knowledge. Furthermore, a survey about impaired microbiome management among university students in Saudi Arabia indicated a significantly higher knowledge level in clinical nutrition students compared to clinical laboratory sciences or public health students [56], though a significantly lower level of probiotic knowledge was previously detected in nutrition students compared to medical students in India [40]. In the present study, being a dietitian, having a PhD level of education as dietitian, or working in an academic/research setting was characterized by higher knowledge scores compared to other groups. For dietitians, working in an academic or research setting was an independent factor for significantly higher current knowledge scoring in all tested sections and overall, based on regression analysis. Furthermore, being a dietitian from the Southern part of Europe was an independent factor for lower scoring in the nutrition as a gut microbiota modulator section and total score compared to other tested regions. These data may provide a glance at the current state of gut microbiota education in dietitians around European regions. Since research about gut microbiota and nutrition-gut microbiome interactions is considered a novel, growing scientific field [57,58], academic exposure of dietitians to current advances in this research area might be more available through MSc or PhD program curricula and not at a pre-graduate level, a fact that could explain the lower current knowledge level of pre-graduate students compared to dietitians participating in this study, and the knowledge discrepancies among regions. Given this context, dietitians working in academic/research areas may follow more closely up-to-date scientific advances, including information about gut microbiota, compared to other nutrition professionals. Paradoxically, dietitians participating from Southern Europe more frequently had a higher educational level (MSc/PhD) compared to Western Europe, or worked more frequently in academia/research compared to the Northern region; these results highlighted the importance of intensifying further life-long learning efforts about gut microbiota. Based on these data and taking into consideration the crucial contribution of dietitians to public awareness about gut microbiota in health [59,60], future educational initiatives about gut microbiota advances are a prerequisite for nutrition experts around Europe, and a more harmonized inclusion of gut microbiome information is necessary in pre-graduate dietetic academic curricula and post-graduate studies among European regions.

The current survey has some limitations. No gender information was available in this study for comparisons; nevertheless, gender-dependent analysis of probiotic knowledge has led to contradictory results in previous studies [9,40,61,62]. Imbalances in representation among European regions were reported, despite efforts to increase response rates from Central-Eastern and Northern Europe during the official circulation of the study. Furthermore, since survey dissemination efforts were mainly implemented through communication channels (e.g., EFAD’s social media and newsletter), but not mailing lists of EFAD’s NDA members, an overlapping of contacts could not be excluded, thus making the response rate of the survey (i.e., the % of people fill out a survey they receive) rather impossible to calculate; a rough estimation based on full participation could be a response rate of around 1% (considering approximately 15,000.00 EFAD social media receivers), which is actually considered quite low for online surveys. Furthermore, the number of full responses to the survey (N = 179) could not form a representative sample of the queried population, calculated according to the established criteria of 95.0% confidence level (CL) and 5.0% margin of error (MoE). Our sample of 179 full responses can suggest conclusions at only 80.0% CL with 5.0% MoE, or at 95.0% CL but with a MoE between 7.0–8.0%. Nevertheless, the survey completion rate of 61.7% was quite acceptable, considering the length of the study questionnaire, giving further validity to the outcomes of the survey. Moreover, pre-testing of the survey helped us to address possible weaknesses and technical issues in the questionnaire before the official launch. Lastly, this study aimed to assess the knowledge and attitudes of both dietitians and pre-graduate dietetic students around Europe regarding different aspects of gut microbiota parameters and nutrition, beyond the use of probiotics and prebiotics. Similar holistic efforts regarding participants [40] or gut microbiota components [55] are internationally scarce to date and further research is necessary.

## 5. Conclusions

In conclusion, this online survey shed some light on the current knowledge and the educational needs of dietitians around Europe in the field of gut microbiota in health, taking into consideration participants’ characteristics. Considerations regarding education about gut microbiome science at the pre-graduate academic level have further emerged. Further research is necessary to elucidate and extrapolate these results at a European level.

## Figures and Tables

**Table 1:** Participant characteristics analysis among European regions (full responses) *.

Participants’ Characteristics	Variable Categories	Central and Eastern Europe	Northern Europe	Southern Europe	Western Europe	Total	Overall *p*
Age group (years)	20–24	3	2	26	2	33	**<0.001**
	25–29	8	3	33	4	48	
	30–34	0	2	17	7	26	
	35–39	1	2	7	4	14	
	40–44	0	1	6	9	16	
	45–49	2	2	5	7	16	
	50–54	0	3	4	10	17	
	55–59	0	2	1	1	4	
	60–65	0	0	0	1	1	
Status	Dietitian	12	14	88	39	153	0.274
	Other professional	0	2	2	4	8	
	Pre-graduate dietetic student	2	1	9	2	14	
Educational level (dietitians) ^†^	Dietitian, BTS	0	0	0	4	4	0.130
	Dietitian, pre-BSc	0	0	4	1	5	
	Dietitian, BSc	6	5	30	17	58	
	Dietitian, MSc	5	8	39	12	64	
	Dietitian, PhD	1	1	15	5	22	
Workplace ^†^	Clinical setting	4	10	20	18	52	0.058
	Community service	0	2	4	2	8	
	Industry	1	0	4	1	6	
	Academia/Research	2	1	26	9	38	
	Freelancer	3	0	29	9	41	
	Other	2	3	7	4	16	
Years in practice as a dietitian ^†^	0–4	8	5	50	7	70	**0.003**
	5–9	2	4	17	10	33	
	10–19	0	3	15	11	29	
	20 or more	2	2	6	11	21	

* For full responses n = 175 due to misclassification of 4 responses—Values are expressed as counts (n) of cases and overall *p* depicts crosstabulation (chi-squared) analysis; Bold values denote statistical significance at the *p* < 0.05 level. ^†^ For educational level and years in practice as a dietitian n = 153, for workplace n = 161.

**Table 2:** Perceived knowledge in each study section and overall estimated perceived knowledge according to participant characteristics (full responses, N = 179) *.

Participants’ Characteristics	Variable Categories	Gut Health in Overall Health	Nutrition as Gut Microbiota Modulator	Probiotics in Health	Prebiotics in Health	Overall Perceived Knowledge
European region ^†^	Central and Eastern (n = 14)	0/6/7/1	0/4/9/1	0/5/8/1	0/3/10/1	0/4/9/1
	Northern (n = 17)	1/6/7/3	1/8/5/3	2/9/4/2	3/8/3/3	1/9/4/3
	Southern (n = 99)	15/46/35/3	13/47/35/4	18/47/30/4	17/49/30/3	14/48/33/4
	Western (n = 45)	8/15/19/3	4/19/19/3	9/22/12/2	10/19/14/2	5/19/19/2
	**overall *p***	0.439	0.260	0.428	**0.022**	0.149
Age group (years)	20–24 (n = 33)	7/16/10/0	7/17/9/0	6/20/7/0	7/20/6/0	7/18/8/0
	25–29 (n = 51)	8/26/14/3	6/20/24/1	8/21/21/1	8/19/23/1	6/22/22/1
	30–34 (n = 27)	1/11/11/4	1/16/5/5	1/17/5/4	2/15/7/3	1/15/6/5
	35–39 (n = 14)	2/5/7/0	1/6/7/0	3/8/3/0	2/6/6/0	1/7/6/0
	40–44 (n = 16)	2/6/6/2	2/6/6/2	3/8/5/0	3/10/2/1	2/7/6/1
	45–49 (n = 16)	2/5/9/0	1/5/9/1	4/4/7/1	4/5/6/1	2/4/9/1
	50–54 (n = 17)	2/5/8/2	0/8/6/3	2/6/6/3	3/5/6/3	1/7/6/3
	55–59 (n = 4)	0/2/2/0	0/3/1/0	2/2/0/0	1/2/1/0	0/3/1/0
	60–65 (n = 1)	0/0/1/0	0/0/1/0	0/0/1/0	0/0/1/0	0/0/1/0
	**overall *p***	0.768	0.235	0.054	0.215	0.147
Status	Dietitian (n = 155)	20/63/61/11	15/67/61/12	21/75/50/9	22/69/55/9	16/69/59/11
	Other professional (n = 9)	1/5/3/0	1/4/4/0	3/4/2/0	3/5/1/0	1/5/3/0
	Pre-graduate dietetic student (n = 15)	3/8/4/0	2/10/3/0	5/7/3/0	5/8/2/0	3/9/3/0
	**overall *p***	0.852	0.712	0.290	0.135	0.546
Educational level (dietitians) ^†^	Dietitian, BTS (n = 4)	1/1/2/0	1/2/1/0	1/2/1/0	1/2/1/0	1/2/1/0
	Dietitian, pre-BSc (n = 5)	2/3/0/0	2/3/0/0	2/3/0/0	3/1/1/0	2/3/0/0
	Dietitian, BSc (n = 58)	11/26/16/5	7/25/21/5	9/26/19/4	10/25/18/5	8/25/20/5
	Dietitian, MSc (n = 65)	5/26/30/4	5/27/29/4	8/33/22/2	7/30/25/3	4/30/28/3
	Dietitian, PhD (n = 23)	1/7/13/2	0/10/10/3	1/11/8/3	1/11/10/1	1/9/10/3
	**overall *p***	**<0.001**	0.487	0.586	0.304	0.327
Workplace ^†^	Clinical setting (n = 52)	7/19/20/6	5/22/20/5	8/26/15/3	8/22/17/5	5/22/20/5
	Community service (n = 8)	1/5/1/1	1/4/2/1	3/3/1/1	3/4/0/1	1/5/1/1
	Industry (n = 6)	2/2/2/0	1/3/2/0	1/2/3/0	1/3/2/0	1/2/3/0
	Academia/ Research (n = 39)	2/11/23/3	1/14/20/4	3/16/17/3	2/16/20/1	1/15/20/3
	Freelancer (n = 42)	7/21/13/1	7/19/14/2	6/22/13/1	7/21/13/1	7/20/14/1
	Other (n = 17)	2/10/5/0	1/9/7/0	3/10/3/1	4/8/4/1	2/10/4/1
	**overall *p***	0.271	**0.046**	0.648	0.295	0.564
Years in practice as a dietitian ^†^	0–4 (n = 71)	12/35/21/3	11/29/29/2	11/33/25/2	13/28/28/2	11/30/28/2
	5–9 (n = 33)	5/12/12/4	3/17/10/3	5/17/8/3	5/17/8/3	3/18/8/4
	10–19 (n = 30)	3/8/16/3	1/13/12/4	4/16/10/0	3/16/10/1	2/13/13/2
	20 or more (n = 21)	0/8/12/1	0/8/10/3	1/9/7/4	1/8/9/3	0/8/10/3
	**overall *p***	0.234	0.393	0.186	0.310	0.190

* Values are expressed as counts (n) of cases of no or poor knowledge/average/good/excellent perceived knowledge for each variable category for the different sections [gut health in overall health, nutrition as a gut microbiota modulator, and the role(s) of probiotics and prebiotics in health] and overall, numbers in parenthesis are counts (n) of cases per variable category, and overall *p* depicts crosstabulation (chi-squared) analysis; Bold values denote statistical significance at the *p* < 0.05 level. ^†^ For European region n = 175, for educational level and years in practice as a dietitian n = 153, for workplace n = 161.

**Table 3:** Current knowledge about the role of gut health in overall health, based on participant characteristics (full responses, N = 179) *.

		Score of Current Knowledge		
Participants’ Characteristics	Variable Categories	Median	Q1–Q3	Overall *p*
European region ^†^	Central and Eastern Europe	18.00	17.25–20.00	0.792
	Northern Europe	19.00	15.00–21.00	
	Southern Europe	18.00	16.00–20.00	
	Western Europe	18.00	16.00–19.00	
Age group (yrs) ^‡^	20–24	18.00	16.00–20.00	0.411
	25–29	18.00	16.00–19.00	
	30–34	18.00	15.00–20.00	
	35–39	19.00	18.75–20.00	
	40–44	17.50	16.00–19.00	
	45–49	17.50	15.25–19.00	
	50–54	20.00	14.00–21.00	
	55–59	19.00	19.00–20.50	
Status	Dietitian	18.00	16.00–20.00	0.173
	Other professional	17.00	15.50–19.00	
	Pre-graduate dietetic student	16.00	15.00–19.00	
Educational level (dietitians) ^†^	Dietitian, BTS	16.50	13.75–18.50	0.139
	Dietitian, pre-BSc	15.00	12.50–19.00	
	Dietitian, BSc	18.00	15.00–20.00	
	Dietitian, MSc	19.00	17.00–20.00	
	Dietitian, PhD	19.00	16.00–20.00	
Workplace ^†^	Clinical setting	18.00	16.25–20.00	**0.035**
	Community service	16.50	10.50–19.00	
	Industry	17.00	11.75–18.00	
	Academia/Research	19.00	17.00–20.00	
	Freelancer	18.00	15.00–20.00	
	Other	18.00	15.50–19.50	
Years in practice as a dietitian ^†^	0–4	18.00	16.00–20.00	0.554
	5–9	18.00	16.00–20.00	
	10–19	19.00	17.00–20.00	
	20 or more	19.00	14.50–20.00	

* Values are expressed as a median (Q1–Q3), and overall *p* depicts nonparametric analysis (Kruskal Wallis H test); Bold values denote statistical significance at the *p* < 0.05 level. ^†^ For educational level and years in practice as a dietitian, n = 155; for workplace, n = 164; for European region, n = 175. ^‡^ For age groups, comparisons for groups <20, 60–65 and >65 years could not be implemented due to few or a complete lack of responses.

**Table 4:** Current knowledge about the role of nutrition as a gut microbiota modulator, based on participant characteristics (full responses, N = 179) *.

		Score of Current Knowledge		
Participants’ Characteristics	Variable Categories	Median	Q1–Q3	Overall *p*
European region ^†^	Central and Eastern Europe	7.00	6.00–9.25	0.168
	Northern Europe	8.00	7.00–9.00	
	Southern Europe	7.00	6.00–8.00	
	Western Europe	8.00	6.00–9.00	
Age group (yrs) ^‡^	20–24	7.00	6.00–9.00	0.348
	25–29	7.00	6.00–9.00	
	30–34	8.00	7.00–8.00	
	35–39	8.50	7.75–9.00	
	40–44	8.00	7.00–9.00	
	45–49	8.50	6.00–9.75	
	50–54	8.00	6.50–9.00	
	55–59	7.50	6.25–8.75	
Status	Dietitian	8.00	6.00–9.00	**0.006**
	Other professional	8.00	7.50–9.00	
	Pre-graduate dietetic student	7.00	5.00–7.00	
Educational level (dietitians) ^†^	Dietitian, BTS	7.50	6.00–9.00	0.078
	Dietitian, pre-BSc	6.00	4.50–7.00	
	Dietitian, BSc	8.00	6.00–9.00	
	Dietitian, MSc	8.00	6.00–9.00	
	Dietitian, PhD	8.00	8.00–9.00	
Workplace ^†^	Clinical setting	8.00	7.00–9.00	**0.034**
	Community service	7.00	4.50–8.00	
	Industry	8.00	5.25–8.75	
	Academia/Research	8.00	8.00–9.00	
	Freelancer	7.00	6.00–9.00	
	Other	7.00	5.50–9.00	
Years in practice as a dietitian ^†^	0–4	8.00	6.00–9.00	0.227
	5–9	7.00	6.00–9.00	
	10–19	8.00	7.00–9.00	
	20 or more	8.00	6.00–9.50	

* Values are expressed as a median (Q1–Q3), and overall *p* depicts nonparametric analysis (Kruskal Wallis H test); bold values denote statistical significance at the *p* < 0.05 level. ^†^ For educational level and years in practice as a dietitian, n = 155; for workplace, n = 164; for European region, n = 175. ^‡^ For age groups, comparisons for groups <20, 60–65 and >65 years could not be implemented due to few or a complete lack of responses.

**Table 5:** Current knowledge about the role of probiotics in health, based on participant characteristics (full responses, N = 179) *.

		Score of Current Knowledge		
Participants’ Characteristics	Variable Categories	Median	Q1–Q3	Overall *p*
European region ^†^	Central and Eastern Europe	5.00	4.75–7.25	0.655
	Northern Europe	6.00	4.00–7.00	
	Southern Europe	5.00	4.00–6.00	
	Western Europe	6.00	4.00–7.00	
Age group (yrs) ^‡^	20–24	5.00	4.00–6.00	0.622
	25–29	6.00	5.00–7.00	
	30–34	6.00	4.00–7.00	
	35–39	6.00	4.00–7.00	
	40–44	5.00	3.25–6.75	
	45–49	5.50	4.25–6.75	
	50–54	5.00	4.00–7.50	
	55–59	5.00	4.25–7.25	
Status	Dietitian	6.00	4.00–7.00	0.137
	Other professional	5.00	5.00–7.00	
	Pre-graduate dietetic student	5.00	3.00–6.00	
Educational level (dietitians) ^†^	Dietitian, BTS	5.00	3.50–8.00	0.183
	Dietitian, pre-BSc	5.00	3.50–5.50	
	Dietitian, BSc	5.00	4.00–7.00	
	Dietitian, MSc	5.00	4.00–7.00	
	Dietitian, PhD	6.00	5.00–7.00	
Workplace ^†^	Clinical setting	5.00	4.00–7.00	0.061
	Community service	5.50	2.75–7.00	
	Industry	3.50	3.00–6.50	
	Academia/Research	6.00	5.00–7.00	
	Freelancer	5.00	5.00–7.00	
	Other	5.00	3.00–6.00	
Years in practice as a dietitian ^†^	0–4	5.00	4.00–7.00	0.738
	5–9	6.00	4.00–7.00	
	10–19	5.00	4.00–7.00	
	20 or more	6.00	5.00–6.50	

* Values are expressed as a median (Q1–Q3), and overall *p* depicts nonparametric analysis (Kruskal Wallis H test). ^†^ For educational level and years in practice as a dietitian, n = 155; for workplace, n = 164; for European region, n = 175. ^‡^ For age groups, comparisons for groups <20, 60–65 and >65 years could not be implemented due to few or a complete lack of responses.

**Table 6:** Current knowledge about the role of prebiotics in health, based on participant characteristics (full responses, N = 179) *.

		Score of Current Knowledge		
Participants’ Characteristics	Variable Categories	Median	Q1–Q3	Overall *p*
European region ^†^	Central and Eastern Europe	3.50	2.00–5.00	0.620
	Northern Europe	3.00	2.00–4.00	
	Southern Europe	3.00	3.00–4.00	
	Western Europe	4.00	3.00–4.50	
Age group (yrs) ^‡^	20–24	3.00	2.00–4.00	0.742
	25–29	3.00	3.00–5.00	
	30–34	3.00	2.00–4.00	
	35–39	3.00	2.75–4.25	
	40–44	3.00	2.25–4.75	
	45–49	4.00	2.25–4.00	
	50–54	4.00	3.00–5.00	
	55–59	3.00	2.25–3.75	
Status	Dietitian	4.00	3.00–4.00	0.108
	Other professional	3.00	2.00–4.00	
	Pre-graduate dietetic student	3.00	2.00–4.00	
Educational level (dietitians) ^†^	Dietitian, BTS	3.50	3.00–4.75	**0.023**
	Dietitian, pre-BSc	3.00	1.50–3.00	
	Dietitian, BSc	3.00	2.00–4.00	
	Dietitian, MSc	4.00	3.00–4.00	
	Dietitian, PhD	4.00	3.00–5.00	
Workplace ^†^	Clinical setting	4.00	3.00–4.00	**0.007**
	Community service	2.50	2.00–4.00	
	Industry	3.00	2.75–3.25	
	Academia/Research	4.00	3.00–5.00	
	Freelancer	3.00	2.00–4.00	
	Other	3.00	2.00–4.00	
Years in practice as a dietitian ^†^	0–4	3.00	2.00–4.00	0.217
	5–9	4.00	3.00–4.00	
	10–19	3.00	2.75–4.00	
	20 or more	4.00	3.00–5.00	

* Values are expressed as a median (Q1–Q3), and overall *p* depicts nonparametric analysis (Kruskal Wallis H test); bold values denote statistical significance at the *p* < 0.05 level. ^†^ For educational level and years in practice as a dietitian, n = 155; for workplace, n = 164; for European region, n = 175. ^‡^ For age groups, comparisons for groups <20, 60–65 and >65 years could not be implemented due to few or a complete lack of responses.

**Table 7:** Total current knowledge of the four sections, based on participant characteristics (full responses, N = 179) *.

		Score of Current Knowledge		
Participants’ Characteristics	Variable Categories	Median	Q1–Q3	Overall *p*
European region ^†^	Central and Eastern Europe	34.00	29.75–41.00	0.601
	Northern Europe	36.00	29.00–40.50	
	Southern Europe	34.00	29.00–38.00	
	Western Europe	36.00	29.00–39.00	
Age group (yrs) ^‡^	20–24	33.00	28.00–37.50	0.677
	25–29	33.00	30.00–38.00	
	30–34	35.00	29.00–38.00	
	35–39	36.50	34.25–39.00	
	40–44	33.00	28.00–37.75	
	45–49	35.50	29.00–38.75	
	50–54	37.00	28.50–41.00	
	55–59	34.50	31.50–37.50	
Status	Dietitian	35.00	30.00–39.00	**0.030**
	Other professional	33.00	29.00–37.00	
	Pre-graduate dietetic student	29.00	28.00–34.00	
Educational level (dietitians) ^†^	Dietitian, BTS	31.00	29.00–39.00	**0.029**
	Dietitian, pre-BSc	27.00	23.50–34.00	
	Dietitian, BSc	35.00	28.00–38.00	
	Dietitian, MSc	36.00	31.50–38.50	
	Dietitian, PhD	38.00	33.00–42.00	
Workplace ^†^	Clinical setting	35.50	30.25–38.00	**0.004**
	Community service	34.00	20.25–36.75	
	Industry	28.00	27.00–33.50	
	Academia/Research	38.00	35.00–41.00	
	Freelancer	33.50	29.00–37.25	
	Other	33.00	26.50–39.00	
Years in practice as a dietitian ^†^	0–4	34.00	28.00–38.00	0.411
	5–9	35.00	30.00–39.00	
	10–19	36.00	31.75–38.25	
	20 or more	38.00	30.00–41.00	

* Values are expressed as a median (Q1–Q3), and overall *p* depicts nonparametric analysis (Kruskal Wallis H test); bold values denote statistical significance at the *p* < 0.05 level. ^†^ For educational level and years in practice as a dietitian, n = 155; for workplace, n = 164; for European region, n = 175. ^‡^ For age groups, comparisons for groups <20, 60–65 and >65 years could not be implemented due to few or a complete lack of responses.

## Data Availability

The data presented in this study are available upon reasonable request from the corresponding author. The data are not publicly available due to legal restrictions by EFAD.

## Supplementary Materials

The following supporting information can be downloaded at: https://www.mdpi.com/article/10.3390/nu16050621/s1, Figure S1: Number of participants (dietitians, pre-graduate dietetic students, other professionals) with full responses according to country origin (N = 179); Figure S2a,b: Current knowledge score according to perceived knowledge classification about the role of gut health in overall health for (a) all participants (N = 179) and (b) for dietitians (n = 155); Figure S3a,b: Current knowledge score according to perceived knowledge classification about the role of nutrition as gut microbiota modulator for (a) all participants (N = 179) and (b) for dietitians (n = 155); Figure S4a,b: Current knowledge score according to perceived knowledge classification about the role of probiotics in health for (a) all participants (N = 179) and (b) for dietitians (n = 155); Figure S5a,b: Current knowledge score according to perceived knowledge classification about the role of prebiotics in health for (a) all participants (N = 179) and (b) for dietitians (n = 155); Figure S6a,b: Total current knowledge score according to overall perceived knowledge for all tested sections for (a) all participants (N = 179) and (b) for dietitians (n = 155); File S1: EFAD surveys-Health through Gut.
