# Supplementary material for: Knowledge of Dietitians on Gut Microbiota in Health—An Online Survey of the European Federation of the Associations of Dietitians (EFAD)
Source: Nutrients. 2024 Feb 23;16(5):621. doi: 10.3390/nu16050621 (PMC10935012; doi:10.3390/nu16050621)
Supplement: Supplementary file 1 [file nutrients-16-00621-s001.zip › Supplementary Figures S1-S6.pdf]

nutrients

Supplementary Materials

Knowledge of Dietitians on Gut Microbiota in Health—An Online Survey of the European Federation of the Associations of Dietitians (EFAD)

Evdokia K. Mitsou [1,*], Christina N. Katsagoni [2,3] and Katarzyna Janiszewska [3]

[1] Department of Nutrition and Dietetics, School of Health Science and Education, Harokopio University, 17671 Athens, Greece
[2] Department of Clinical Nutrition, Agia Sofia Children's Hospital, 11527 Athens, Greece; christina.katsagoni@gmail.com
[3] The European Federation of the Associations of Dietitians (EFAD), Gooimeer 4-15, 1411 DC Naarden, The Netherlands; katarzyna.janiszewska@efad.org
* Correspondence: emitsou@hua.gr; Tel.: +30-210-9549301

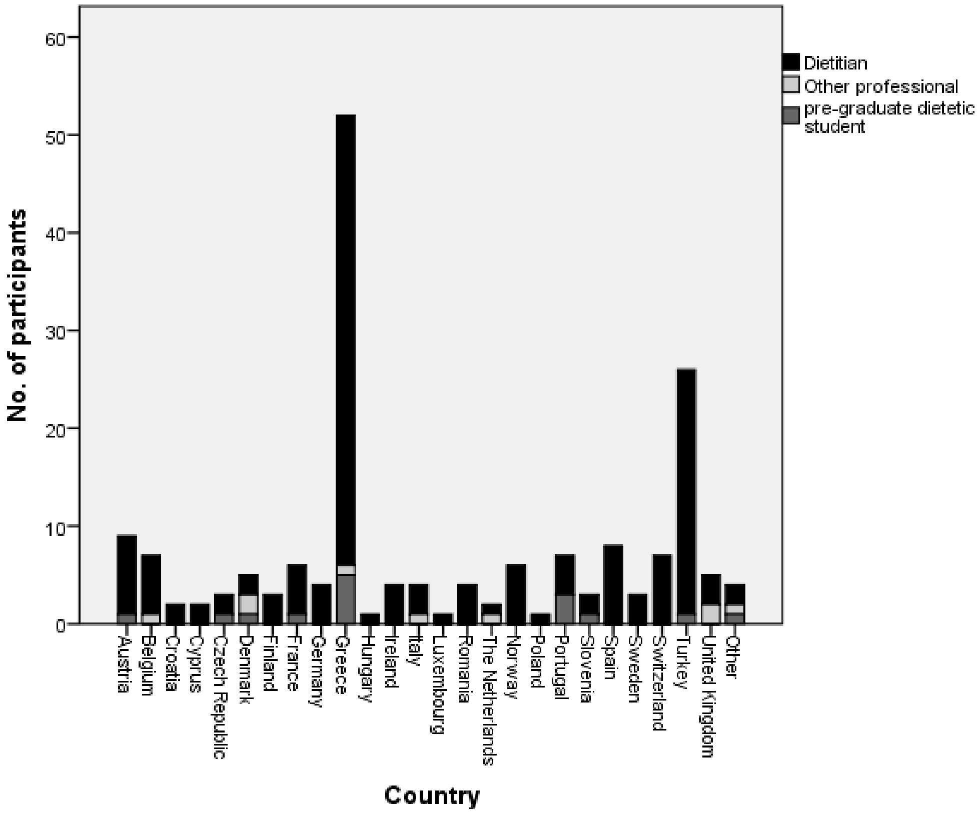

Figure S1. Number of participants (dietitians, pre-graduate dietetic students, other professionals) with full responses according to country origin (N=179).

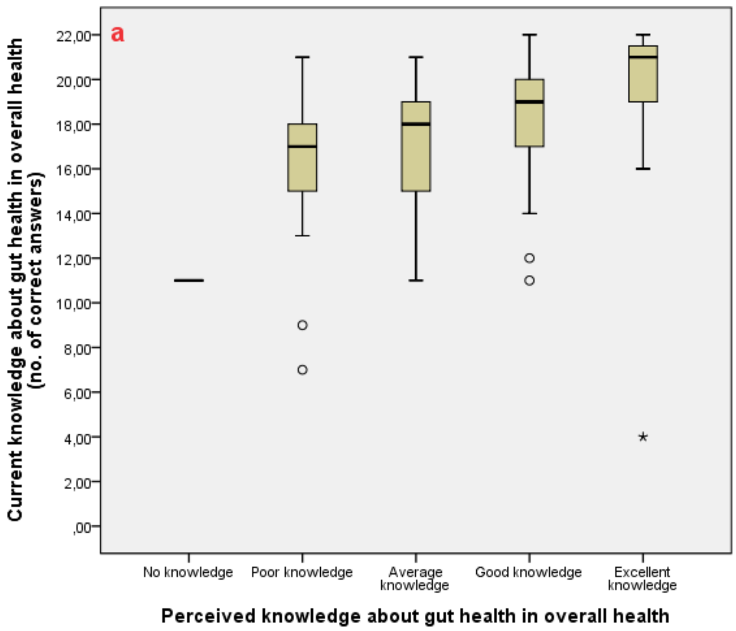

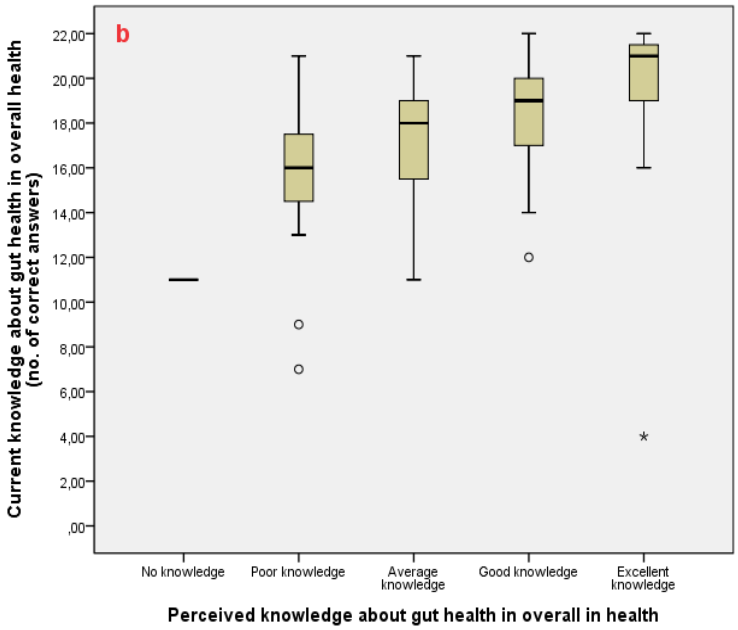

Figure S2. (**a**,**b**) Current knowledge score according to perceived knowledge classification about the role of gut health in overall health for (a) all participants (N=179) (Kruskal-Wallis test, overall $p<0.001$; Spearman's rho 0.353, $p<0.001$) and (b) for dietitians (n=155) (Kruskal-Wallis test, overall $p<0.001$; Spearman's rho 0.372, $p<0.001$); Score values (no. of correct responses out of 22 questions) are illustrated as boxplots.

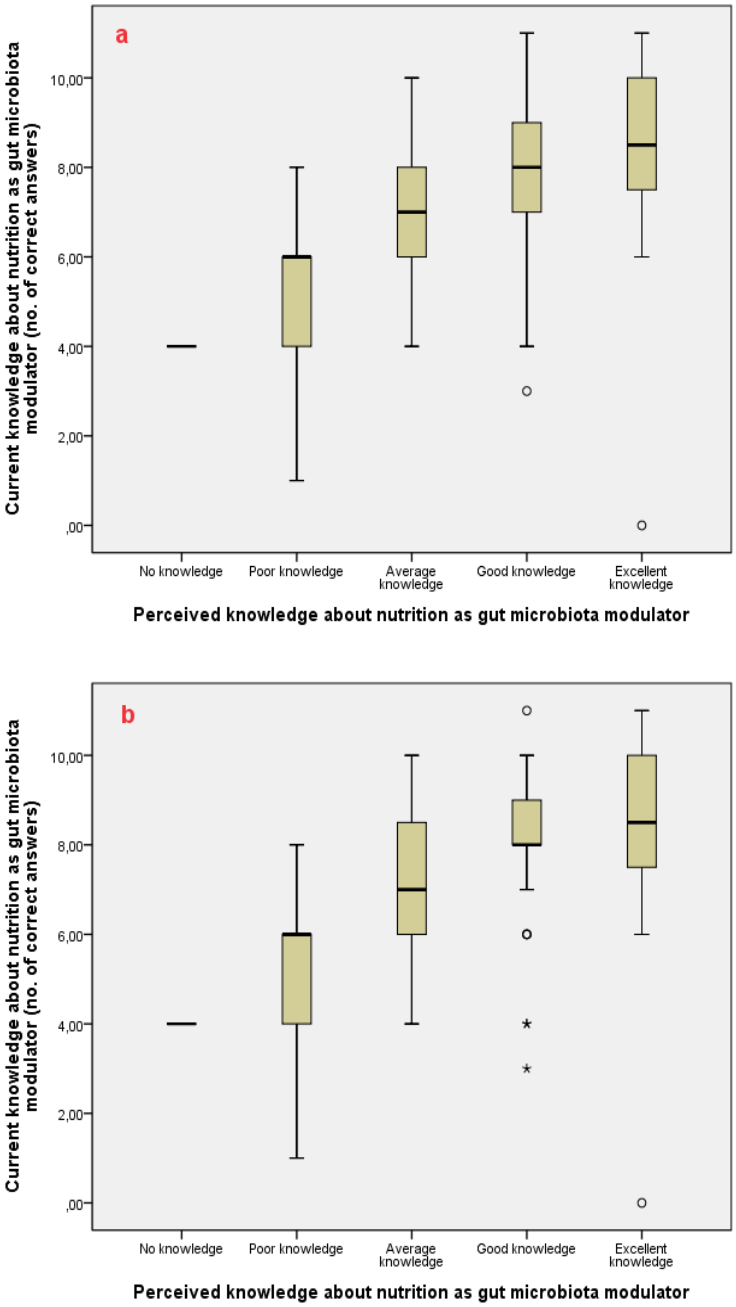

Figure S3. (**a**,**b**) Current knowledge score according to perceived knowledge classification about the role of nutrition as gut microbiota modulator for (a) all participants (N=179) (Kruskal-Wallis test, overall $p<0.001$; Spearman's rho 0.454, $p<0.001$) and (b) for dietitians (n=155) (Kruskal-Wallis test, overall $p<0.001$; Spearman's rho 0.447, $p<0.001$); Score values (no. of correct responses out of 11 questions) are illustrated as boxplots.

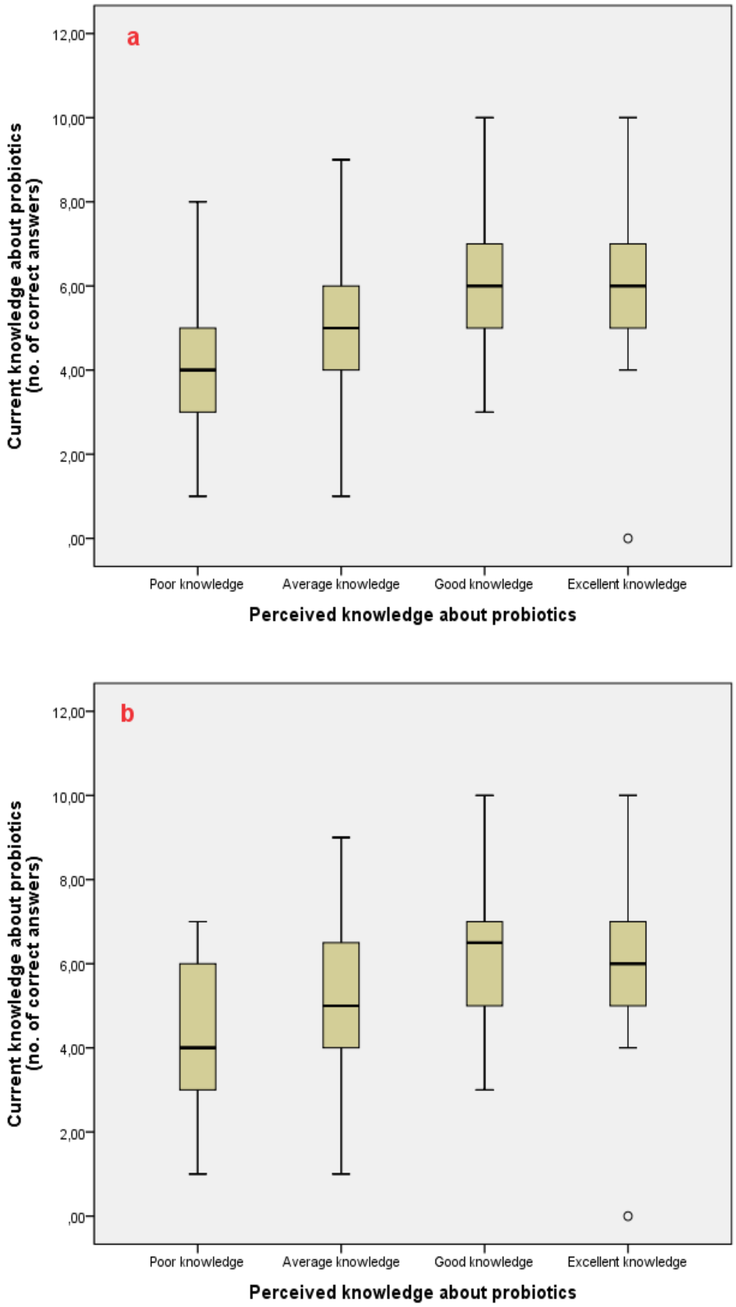

Figure S4. (**a**,**b**) Current knowledge score according to perceived knowledge classification about the role of probiotics in health for (a) all participants (N=179) (Kruskal-Wallis test, overall p<0.001; Spearman's rho 0.362, p<0.001) and (b) for dietitians (n=155) (Kruskal-Wallis test, overall p<0.001; Spearman's rho 0.361, p<0.001); Score values (no. of correct response out of 12 questions) are illustrated as boxplots.

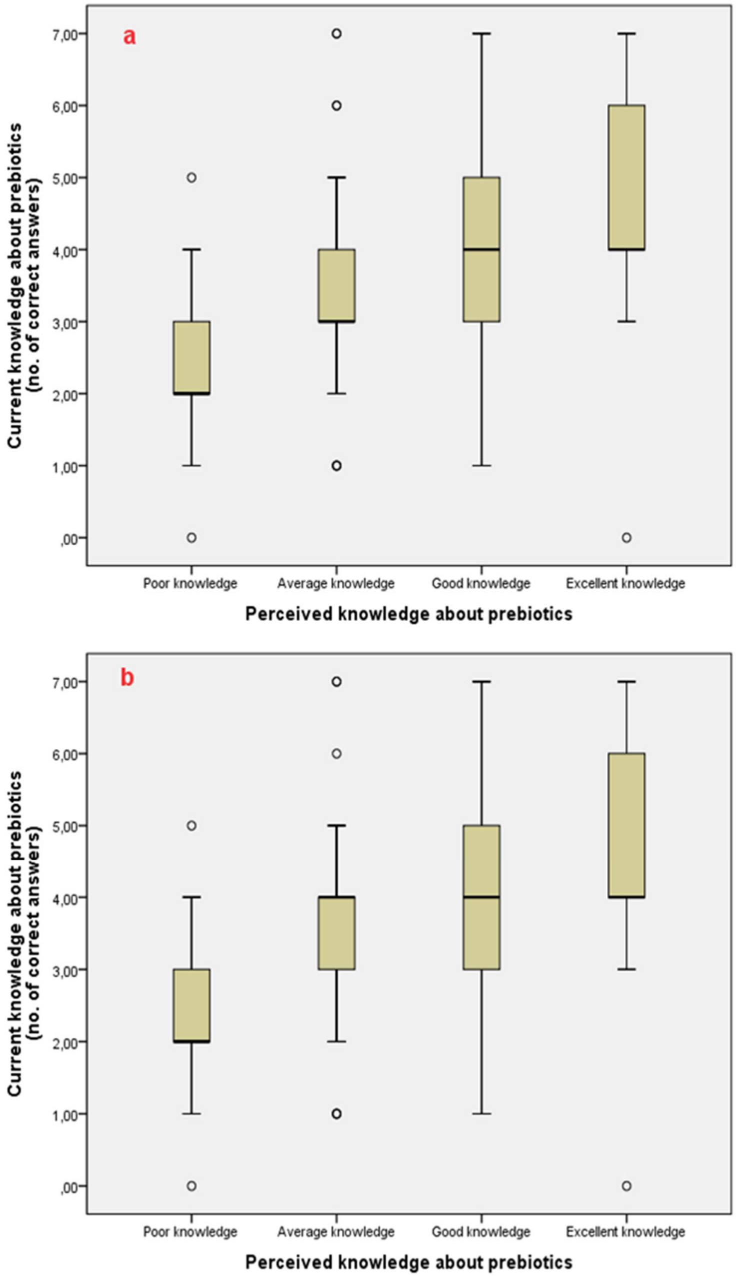

Figure S5. (**a**,**b**) Current knowledge score according to perceived knowledge classification about the role of prebiotics in health for (a) all participants (N=179) (Kruskal-Wallis test, overall p<0.001; Spearman's rho 0.351, p<0.001) and (b) for dietitians (n=155) (Kruskal-Wallis test, overall p<0.001; Spearman's rho 0.347, p<0.001); Score values (no. of correct response out of seven questions) are illustrated as boxplots.

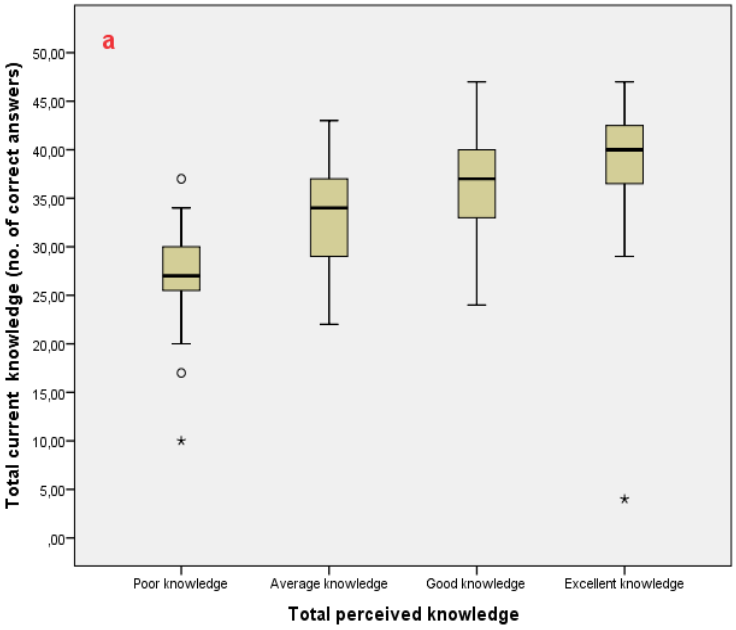

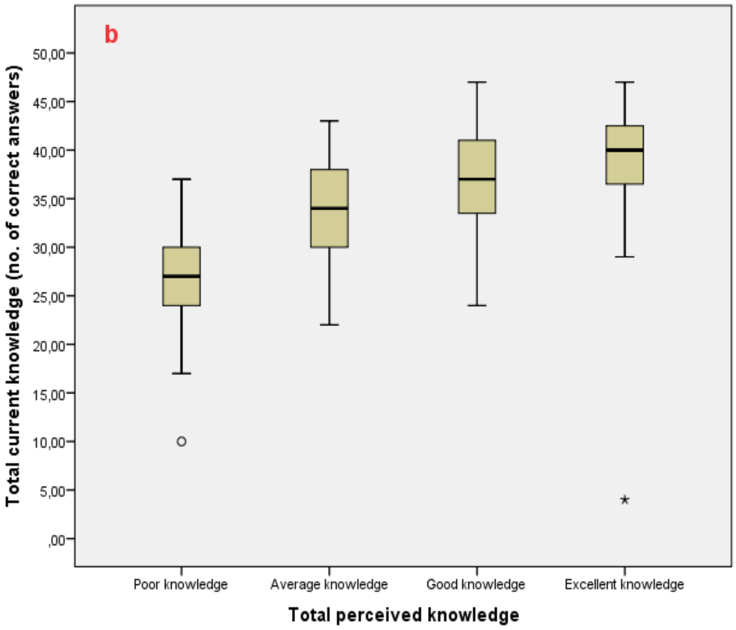

Figure S6. (**a**,**b**) Total current knowledge score according to overall perceived knowledge for all tested sections for (a) all participants (N=179) (Kruskal-Wallis test, overall p<0.001; Spearman's rho 0.459, p<0.001) and (b) for dietitians (n=155) (Kruskal-Wallis test, overall p<0.001; Spearman's rho 0.460, p<0.001); Score values (no. of correct responses out of 52 questions) are illustrated as boxplots.
