# Supplementary material for: Knowledge of Dietitians on Gut Microbiota in Health—An Online Survey of the European Federation of the Associations of Dietitians (EFAD)
Source: Nutrients. 2024 Feb 23;16(5):621. doi: 10.3390/nu16050621 (PMC10935012; doi:10.3390/nu16050621)
Supplement: Supplementary file 1 [file nutrients-16-00621-s001.zip › Supplementary Materials File S1-EFAD surveys-Health through Gut.pdf]

Health Through Gut Survey

Thank you for your interest in taking the Health Through Gut Survey of the European Federation of the Associations of Dietitians (EFAD)

The purpose of the survey is to assess the level of knowledge and possible educational needs of dietitians and pre-graduate dietetic students across Europe about health through gut, microorganisms of the gut and the role of nutrition and dietary choices as modulators of the gut ecosystem. The analysis of the results of the survey will further support EFAD to develop online educational programs about health through gut, focusing on dissemination of high-quality knowledge and addressing specific educational needs. That is why EFAD needs your opinion and knowledge in this field.

This a survey, not a test and since all information is anonymous, it will not be possible to provide feedback afterwards.

The survey will take approximately 15 minutes to complete.

Your participation in this study is completely voluntary and you will not be paid any allowance. There are no foreseeable risks associated with this survey. However, if you feel uncomfortable answering any question, you can withdraw from the survey at any time and without giving a reason. This would not affect your legal rights.

Best practice with ethical and legal practice will be complied with. Personal identification information (IP address) will be collected automatically by EFAD to avoid multiple responses from the same participant – use of cookies for access control will be also applied. Declaration of your country will be used for assessing survey response rate around Europe and for statistical purposes only. Age groups of participants will be stored and reported only in aggregated form for statistical purposes. All rules on the protection of individuals regarding the processing of personal data are followed in compliance with the requirements of GDPR and the Data Protection Act 2018. Privacy policy regarding your personal data is available at https://www.nottingham.ac.uk/utilities/privacy/privacy.aspx (https://www.nottingham.ac.uk/utilities/privacy/privacy.aspx).

Your responses will remain anonymous and none of the responses will be connected to identifying information. All your responses will be kept confidential, secured within the University of Nottingham, with a sharing agreement with the EFAD. Your responses will be used only for statistical purposes and will be reported only in aggregated form. Data from this survey will further help EFAD to develop online educational programs.

By pressing **'Next'** you are consenting to participate in this survey under the terms outlined above.

This survey has undergone ethical review and approval by the School of Sociology and Social Policy Research Ethics Committee from the University of Nottingham. If you have a concern about any aspect of this survey, you should ask to speak with the main investigator Fiona McCullough, Associate Professor of Dietetics, School of Biosciences, who will answer your questions (Fiona.McCullough@nottingham.ac.uk (mailto:Fiona.McCullough@nottingham.ac.uk))

There are 77 questions in this survey.

Country

*

❗ Choose one of the following answers
Please choose **only one** of the following:

- ◯ Austria
- ◯ Belgium
- ◯ Croatia
- ◯ Cyprus
- ◯ Czech Republic
- ◯ Denmark
- ◯ Finland
- ◯ France
- ◯ Germany
- ◯ Greece
- ◯ Hungary
- ◯ Iceland
- ◯ Ireland
- ◯ Israel
- ◯ Italy
- ◯ Lithuania
- ◯ Luxembourg
- ◯ Romania
- ◯ The Netherlands
- ◯ Norway
- ◯ Poland
- ◯ Portugal
- ◯ Slovenia
- ◯ Spain
- ◯ Sweden
- ◯ Switzerland
- ◯ Turkey

◯ United Kingdom

◯ Other [ ]

Age (years) *

❗ Choose one of the following answers
Please choose **only one** of the following:

◯ < 20
◯ 20 - 24
◯ 25 - 29
◯ 30 - 34
◯ 35 - 39
◯ 40 - 44
◯ 45 - 49
◯ 50 - 54
◯ 55 - 59
◯ 60 - 65
◯ > 65

Educational level *

❗ Choose one of the following answers
Please choose **only one** of the following:

◯ pre-graduate dietetic student
◯ Dietitian, BTS (Brevet de technicien supérieur)
◯ Dietitian, pre-BSc
◯ Dietitian, Bachelor's Degree (BSc)
◯ Dietitian, Master's Degree (MSc)
◯ Dietitian, Doctorate Degree (PhD)
◯ I am a professional, but not a dietitian.

Workplace *

Only answer this question if the following conditions are met:
Answer was 'Dietitian, BTS (Brevet de technicien supérieur)' *or* 'Dietitian, Master's Degree (MSc)' *or* 'Dietitian, pre-BSc' *or* 'Dietitian, Bachelor's Degree (BSc)' *or* 'Dietitian, Doctorate Degree (PhD)' *or* 'I am a professional, but not a dietitian.' at question '3 [a003]' (Educational level)

❗ Choose one of the following answers
❗ If you choose 'Other:' please also specify your choice in the accompanying text field.
Please choose **only one** of the following:

◯ clinical setting
◯ community service
◯ industry
◯ academia/research
◯ freelancer
◯ Other ________

Years in practice as a dietitian *

Only answer this question if the following conditions are met:
Answer was 'Dietitian, BTS (Brevet de technicien supérieur)' *or* 'Dietitian, pre-BSc' *or* 'Dietitian, Bachelor's Degree (BSc)' *or* 'Dietitian, Master's Degree (MSc)' *or* 'Dietitian, Doctorate Degree (PhD)' at question '3 [a003]' (Educational level)

❗ Choose one of the following answers
Please choose **only one** of the following:

◯ 0 - 4
◯ 5 - 9
◯ 10 - 19
◯ 20 or more

How do you rate your knowledge about the role of gut health in overall health? *

❗ Choose one of the following answers
Please choose **only one** of the following:

- ◯ No knowledge
- ◯ Poor knowledge
- ◯ Average knowledge
- ◯ Good knowledge
- ◯ Excellent knowledge

Gut health impacts the overall health.

❗ Choose one of the following answers
Please choose **only one** of the following:

- ◯ True
- ◯ False
- ◯ I don't know

What is true about gut?

❗ Choose one of the following answers
Please choose **only one** of the following:

- ◯ gut is the largest immune and endocrine organ in our body
- ◯ gut interconnects highly with other body organs
- ◯ gut may affect metabolic and mental health
- ◯ all the above
- ◯ I don't know

Gut barrier function serves as a mechanical, immunological and biological line of health defence.

❶ Choose one of the following answers
Please choose **only one** of the following:

- ◯ True
- ◯ False
- ◯ I don't know

Gut barrier dysfunction is observed only in acute disease states.

❶ Choose one of the following answers
Please choose **only one** of the following:

- ◯ True
- ◯ False
- ◯ I don't know

Gut-brain axis disruption is often present in irritable bowel syndrome and autism spectrum disorders.

❶ Choose one of the following answers
Please choose **only one** of the following:

- ◯ True
- ◯ False
- ◯ I don't know

Everybody has a unique set of gut microorganisms, known as gut microbiota.

❶ Choose one of the following answers
Please choose **only one** of the following:

- True
- False
- I don't know

Gut microbiota composition is stable from birth until death.

❶ Choose one of the following answers
Please choose **only one** of the following:

- True
- False
- I don't know

Most of the microorganisms in our body are in the large bowel.

❶ Choose one of the following answers
Please choose **only one** of the following:

- True
- False
- I don't know

Gut microbiota consists of bacteria but also fungi, archaea, viruses.

❗ Choose one of the following answers
Please choose **only one** of the following:

- ◯ True
- ◯ False
- ◯ I don't know

Gut microorganisms are mainly pathogens and should be controlled by antibiotics.

❗ Choose one of the following answers
Please choose **only one** of the following:

- ◯ True
- ◯ False
- ◯ I don't know

Gut microorganisms are mainly symbiotic and promote health.

❗ Choose one of the following answers
Please choose **only one** of the following:

- ◯ True
- ◯ False
- ◯ I don't know

Which factor(s) can influence the shaping of gut microbiota composition?

❗ Choose one of the following answers
Please choose **only one** of the following:

- ◯ Our genome
- ◯ Food and nutrients
- ◯ Drugs
- ◯ Fecal Microbiota Transfer
- ◯ Physical exersice
- ◯ All of them
- ◯ None of them
- ◯ I don't know

Overall health status can be related to diversity of gut microbiota.

❗ Choose one of the following answers
Please choose **only one** of the following:

- ◯ True
- ◯ False
- ◯ I don't know

Dysbiosis is an imbalance of gut microbial communities, usually observed in obesity and diabetes.

❗ Choose one of the following answers
Please choose **only one** of the following:

- ◯ True
- ◯ False
- ◯ I don't know

Dysbiosis can include a loss of overall gut microbial diversity, a drop in beneficial bacteria and/or an overgrowth of potential pathogens.

❗ Choose one of the following answers
Please choose **only one** of the following:

- ◯ True
- ◯ False
- ◯ I don't know

Certain gut microorganisms and metabolites can trigger inflammation within and beyond gut in our body.

❗ Choose one of the following answers
Please choose **only one** of the following:

- ◯ True
- ◯ False
- ◯ I don't know

Crohn’s disease and colon cancer are connected to gut microbiota.

❗ Choose one of the following answers
Please choose **only one** of the following:

- ◯ True
- ◯ False
- ◯ I don't know

Immune response to Covid-19 infection might be influenced by gut microbiota.

❗ Choose one of the following answers
Please choose **only one** of the following:

- ◯ True
- ◯ False
- ◯ I don't know

Metabolic potential of gut microbiota surpasses that of the human host.

❗ Choose one of the following answers
Please choose **only one** of the following:

- ◯ True
- ◯ False
- ◯ I don't know

A beneficial metabolite produced from nutrients by gut microbiota is:

❗ Choose one of the following answers
Please choose **only one** of the following:

- ◯ Methane
- ◯ Hydrogen Sulfide (H_2S)
- ◯ Ammonia
- ◯ Butyrate
- ◯ I don't know

Transplantation of gut microorganisms is an evidence-based treatment for:

❗ Choose one of the following answers
Please choose **only one** of the following:

- ◯ Obesity and diabetes
- ◯ Colon cancer
- ◯ Crohn's disease and ulcerative colitis
- ◯ Clostridium difficile infection
- ◯ I don't know

Where do we routinely test gut microbes?

❗ Choose one of the following answers
Please choose **only one** of the following:

- ◯ in blood
- ◯ in urine
- ◯ in stool
- ◯ in biopsies
- ◯ I don't know

How do you rate your knowledge about the role of nutrition as a modulator of gut microbiota? *

❗ Choose one of the following answers
Please choose **only one** of the following:

- ◯ No knowledge
- ◯ Poor knowledge
- ◯ Average knowledge
- ◯ Good knowledge
- ◯ Excellent knowledge

How do you rate your knowledge about the role of probiotics in health?
*****

❗ Choose one of the following answers
Please choose **only one** of the following:

- ◯ No knowledge
- ◯ Poor knowledge
- ◯ Average knowledge
- ◯ Good knowledge
- ◯ Excellent knowledge

How do you rate your knowledge about the role of prebiotics in health?
*****

❗ Choose one of the following answers
Please choose **only one** of the following:

- ◯ No knowledge
- ◯ Poor knowledge
- ◯ Average knowledge
- ◯ Good knowledge
- ◯ Excellent knowledge

Dietary modifications can cause changes to gut microbiota within 24 hours.

❗ Choose one of the following answers
Please choose **only one** of the following:

- ◯ True
- ◯ False
- ◯ I don't know

Different long-term dietary patterns result in different gut microbiota profiles.

❶ Choose one of the following answers
Please choose **only one** of the following:

- ◯ True
- ◯ False
- ◯ I don't know

The more diverse the diet, the more diverse the gut microbiota.

❶ Choose one of the following answers
Please choose **only one** of the following:

- ◯ True
- ◯ False
- ◯ I don't know

A western type of diet and lifestyle is associated with more diverse gut microbiota.

❶ Choose one of the following answers
Please choose **only one** of the following:

- ◯ True
- ◯ False
- ◯ I don't know

Dietary carbohydrates are major drivers of gut microbiota characteristics.

❗ Choose one of the following answers
Please choose **only one** of the following:

- True
- False
- I don't know

Gut microorganisms may affect the ability to extract energy from food.

❗ Choose one of the following answers
Please choose **only one** of the following:

- True
- False
- I don't know

Industrialised food products we consume are sterile, with no bacterial load.

❗ Choose one of the following answers
Please choose **only one** of the following:

- True
- False
- I don't know

Foods carry always pathogens and need decontamination before consumption.

❶ Choose one of the following answers
Please choose **only one** of the following:

- ◯ True
- ◯ False
- ◯ I don't know

Foods may contain beneficial microorganisms that exert health effects.

❶ Choose one of the following answers
Please choose **only one** of the following:

- ◯ True
- ◯ False
- ◯ I don't know

Yogurt, bread, beer and wine are fermented foods with live microorganisms present.

❶ Choose one of the following answers
Please choose **only one** of the following:

- ◯ True
- ◯ False
- ◯ I don't know

There is a Recommended Dietary Allowance (RDA) for ingested microorganisms.

❗ Choose one of the following answers
Please choose **only one** of the following:

- ◯ True
- ◯ False
- ◯ I don't know

Choose the definition of probiotics: *

❗ Choose one of the following answers
Please choose **only one** of the following:

- ◯ Probiotics are all microorganisms consumed with foods and dietary supplements.
- ◯ Probiotics are fermentable ingredients that promote the growth of the gut microbiota.
- ◯ Probiotics are live microorganisms, that when administered in adequate amounts, confer a health benefit to the host.
- ◯ Probiotics are lactobacilli and bifidobacteria, that when administered in adequate amounts, confer a health benefit to the host.
- ◯ I don’t know the definition.

Choose the definition of prebiotics: *

Choose one of the following answers
Please choose **only one** of the following:

- Prebiotics are live microorganisms, that when administered in adequate amounts, confer a health benefit to the host.
- Prebiotics are combinations of microorganisms and fibers that promote health through gut.
- Prebiotics are dietary fibers that are fermentable and promote the growth of the gut microbiota.
- Prebiotics are substrates that are selectively utilized by host microorganisms conferring a health benefit.
- I don’t know the definition.

Probiotics should colonize the gut to exert beneficial effects.

Choose one of the following answers
Please choose **only one** of the following:

- True
- False
- I don't know

Probiotics are washed away from gut when administration is stopped.

Choose one of the following answers
Please choose **only one** of the following:

- True
- False
- I don't know

Probiotics have strain-specific effects.

❗ Choose one of the following answers
Please choose **only one** of the following:

- True
- False
- I don't know

Probiotics are more efficient as food than dietary supplements.

❗ Choose one of the following answers
Please choose **only one** of the following:

- True
- False
- I don't know

Probiotics are more efficient in larger quantities.

❗ Choose one of the following answers
Please choose **only one** of the following:

- True
- False
- I don't know

Probiotics are more efficient when they contain different microorganisms.

❗ Choose one of the following answers
Please choose **only one** of the following:

- ◯ True
- ◯ False
- ◯ I don't know

All fermented foods are considered as probiotics.

❗ Choose one of the following answers
Please choose **only one** of the following:

- ◯ True
- ◯ False
- ◯ I don't know

For most healthy people, probiotics are generally safe to consume.

❗ Choose one of the following answers
Please choose **only one** of the following:

- ◯ True
- ◯ False
- ◯ I don't know

Some probiotic strains of lactobacilli and yeasts are commonly used for prevention of antibiotic-associated diarrhea.

❗ Choose one of the following answers
Please choose **only one** of the following:

- ◯ True
- ◯ False
- ◯ I don't know

Many European countries have national dietary guidelines or recommendations about probiotics.

❗ Choose one of the following answers
Please choose **only one** of the following:

- ◯ True
- ◯ False
- ◯ I don't know

Probiotics have many health claims authorised in European Union.

❗ Choose one of the following answers
Please choose **only one** of the following:

- ◯ True
- ◯ False
- ◯ I don't know

Prebiotics can be found naturally in foods (e.g. onions, bananas, chicory root, human milk).

❗ Choose one of the following answers
Please choose **only one** of the following:

- ◯ True
- ◯ False
- ◯ I don't know

All dietary fibers are prebiotics.

❗ Choose one of the following answers
Please choose **only one** of the following:

- ◯ True
- ◯ False
- ◯ I don't know

There is a Recommended Dietary Allowance (RDA) for prebiotics.

❗ Choose one of the following answers
Please choose **only one** of the following:

- ◯ True
- ◯ False
- ◯ I don't know

High amounts of prebiotics can cause some gas or bloating.

❗ Choose one of the following answers
Please choose **only one** of the following:

- ◯ True
- ◯ False
- ◯ I don't know

Psyllium is the best known prebiotic with evidence-based health effects.

❗ Choose one of the following answers
Please choose **only one** of the following:

- ◯ True
- ◯ False
- ◯ I don't know

Prebiotics have many health claims authorised in European Union.

❗ Choose one of the following answers
Please choose **only one** of the following:

- ◯ True
- ◯ False
- ◯ I don't know

Do you believe that probiotics may be beneficial in the following health situations?
*

Please choose the appropriate response for each item:

	strongly don't believe	don't believe	neutral (don't know)	believe	strongly believe
**Lactose digestion**	○	○	○	○	○
**Constipation**	○	○	○	○	○
**Antibiotic-associated diarrhea**	○	○	○	○	○
**Clostridium difficile infection during antibiotic treatment**	○	○	○	○	○
**Crohn's disease**	○	○	○	○	○
**Pouchitis**	○	○	○	○	○
**Necrotizing enterocolitis in premature infants**	○	○	○	○	○
**Irritable Bowel Syndrome**	○	○	○	○	○
**Allergies**	○	○	○	○	○
**Obesity**	○	○	○	○	○
**Heart health**	○	○	○	○	○
**Mental health/stress**	○	○	○	○	○

Do you believe that prebiotics may be beneficial in the following health situations?
*

Please choose the appropriate response for each item:

	strongly don't believe	don't believe	neutral (don't know)	believe	strongly believe
**Improvement of bowel habits**	◯	◯	◯	◯	◯
**Mineral absorption**	◯	◯	◯	◯	◯
**Immune system modulation**	◯	◯	◯	◯	◯
**Risk of allergy**	◯	◯	◯	◯	◯
**Satiety**	◯	◯	◯	◯	◯
**Blood lipid and glucose levels**	◯	◯	◯	◯	◯

Do you believe that probiotics and prebiotics have a place in nutritional practice?
*

❗ Choose one of the following answers
Please choose **only one** of the following:

◯ strongly don't believe
◯ don't believe
◯ neutral (don't know)
◯ believe
◯ strongly believe

Do you believe that fermented foods with live microbial cultures, like yogurt, should be a part of Food Based Dietary Guidelines in your country?
*

❶ Choose one of the following answers
Please choose **only one** of the following:

- ◯ strongly don't believe
- ◯ don't believe
- ◯ neutral (don't know)
- ◯ believe
- ◯ strongly believe

Based on your daily practice, do you believe that manipulation of gut microbiota by dietary choices is of great importance for nutritional counseling?
*

Only answer this question if the following conditions are met:
Answer was 'Dietitian, BTS (Brevet de technicien supérieur)' *or* 'Dietitian, pre-BSc' *or* 'Dietitian, Bachelor's Degree (BSc)' *or* 'Dietitian, Master's Degree (MSc)' *or* 'Dietitian, Doctorate Degree (PhD)' at question '3 [a003]' (Educational level)

❶ Choose one of the following answers
Please choose **only one** of the following:

- ◯ strongly don't believe
- ◯ don't believe
- ◯ neutral (don't know)
- ◯ believe
- ◯ strongly believe

Based on your current knowledge, are you confident to apply gut microbiota information in your daily dietary practice?
*

Only answer this question if the following conditions are met:
Answer was 'Dietitian, BTS (Brevet de technicien supérieur)' *or* 'Dietitian, pre-BSc' *or* 'Dietitian, Bachelor's Degree (BSc)' *or* 'Dietitian, Master's Degree (MSc)' *or* 'Dietitian, Doctorate Degree (PhD)' at question '3 [a003]' (Educational level)

Choose one of the following answers
Please choose **only one** of the following:

- not at all confident
- not very confident
- neutral
- somewhat confident
- very confident

Do you believe that commercially available gut microbiota test kits could be helpful in your daily dietary practice and recommendations?
*

Only answer this question if the following conditions are met:
Answer was 'Dietitian, BTS (Brevet de technicien supérieur)' *or* 'Dietitian, pre-BSc' *or* 'Dietitian, Bachelor's Degree (BSc)' *or* 'Dietitian, Master's Degree (MSc)' *or* 'Dietitian, Doctorate Degree (PhD)' at question '3 [a003]' (Educational level)

Choose one of the following answers
Please choose **only one** of the following:

- strongly don't believe
- don't believe
- neutral (don't know)
- believe
- strongly believe

Do you advise the use of probiotics/prebiotics to patients or your close ones? *

Only answer this question if the following conditions are met:
Answer was 'Dietitian, BTS (Brevet de technicien supérieur)' *or* 'Dietitian, pre-BSc' *or* 'Dietitian, Bachelor's Degree (BSc)' *or* 'Dietitian, Master's Degree (MSc)' *or* 'Dietitian, Doctorate Degree (PhD)' at question '3 [a003]' (Educational level)

❗ Choose one of the following answers
Please choose **only one** of the following:

- ◯ never
- ◯ rarely
- ◯ sometimes
- ◯ often
- ◯ always

If you advise the use of probiotics/prebiotics, do you recommend intake through food or through dietary supplements? *

Only answer this question if the following conditions are met:
Answer was 'rarely' *or* 'sometimes' *or* 'often' *or* 'always' at question '69 [h004]' (Do you advise the use of probiotics/prebiotics to patients or your close ones?)

❗ Choose one of the following answers
Please choose **only one** of the following:

- ◯ Food
- ◯ Supplements
- ◯ Both

Based on the existing guidelines, in which health conditions a dietitian should recommend the use of probiotics? *

Only answer this question if the following conditions are met:
Answer was 'Dietitian, BTS (Brevet de technicien supérieur)' *or* 'Dietitian, pre-BSc' *or* 'Dietitian, Bachelor's Degree (BSc)' *or* 'Dietitian, Master's Degree (MSc)' *or* 'Dietitian, Doctorate Degree (PhD)' at question '3 [a003]' (Educational level)

Check all that apply
Please choose **all** that apply:

- [ ] prevention of antibiotic-associated diarrhea
- [ ] relief of mild gastrointestinal symptoms
- [ ] lactose maldigestion
- [ ] pouchitis
- [ ] weight management
- [ ] control of glucose and cholesterol levels
- [ ] Other: ______

What is your source(s) of information about gut microbiota/health through gut? *

Check all that apply
Please choose **all** that apply:

- [ ] Academic classes/lectures
- [ ] Research publications
- [ ] Conferences
- [ ] Seminars/Webinars
- [ ] Media (TV-podcasts)
- [ ] Social media/Blogs
- [ ] Representatives from companies
- [ ] Never informed before
- [ ] Other: ______

Would you be interested in attending webinars/e-courses in the future focusing on health through gut?
*

Choose one of the following answers
Please choose **only one** of the following:

- not at all interested
- not very interested
- neutral
- somewhat interested
- very interested

What kind of e-learning format(s) would be more appealing to you in a future webinar/e-course about health through gut? *

Only answer this question if the following conditions are met:
Answer was 'neutral' *or* 'somewhat interested' *or* 'very interested' at question '73 [i002]' (Would you be interested in attending webinars/e-courses in the future focusing on health through gut?)

Check all that apply
Please choose **all** that apply:

- Slides with voice over
- Educational videos
- Video interviews with experts
- Infographics
- Short articles
- Case studies
- Other:

What could be your area(s) of interest in a future webinar/e-course about health through gut? *

Only answer this question if the following conditions are met:
Answer was 'neutral' *or* 'somewhat interested' *or* 'very interested' at question '73 [i002]' (Would you be interested in attending webinars/e-courses in the future focusing on health through gut?)

❗ Check all that apply
Please choose **all** that apply:

- ☐ Gut functions and interconnections with other organs
- ☐ Gut microbiota composition and metabolism in health and disease
- ☐ Dietary patterns and gut microbiota
- ☐ Use of probiotics and prebiotics in clinical practice
- ☐ Personalized nutrition through gut microbiota manipulation
- ☐ Other: ____________

What factor might have discouraged you from getting trained in the field of health through gut in the past?

❗ Choose one of the following answers
Please choose **only one** of the following:

- ◯ Lack of interest in the topic
- ◯ Perceived complexity of gut microbiota terminology and functions
- ◯ Perceived complexity in applying relevant knowledge in dietetics
- ◯ Conference fees/Travel expenses
- ◯ Not discouraged
- ◯ Other ____________

How much money on average do you spend annually for nutrition training (in any field)?

❗ Choose one of the following answers
Please choose **only one** of the following:

- ◯ More than 1000 €
- ◯ 500 - 1000 €
- ◯ 100 - 499 €
- ◯ Less than 100 €
- ◯ I don't usually undertake any training.

Submit your survey.
Thank you for completing this survey.
